# Maternal pomegranate juice intake and brain structure and function in infants with intrauterine growth restriction: A randomized controlled pilot study

**DOI:** 10.1371/journal.pone.0219596

**Published:** 2019-08-21

**Authors:** Lillian G. Matthews, Christopher D. Smyser, Sara Cherkerzian, Dimitrios Alexopoulos, Jeanette Kenley, Methodius G. Tuuli, D. Michael Nelson, Terrie E. Inder

**Affiliations:** 1 Department of Pediatric Newborn Medicine, Brigham and Women’s Hospital, Harvard Medical School, Boston, Massachusetts, United States of America; 2 Department of Pediatrics, Washington University, Saint Louis, Missouri, United States of America; 3 Department of Neurology, Washington University, Saint Louis, Missouri, United States of America; 4 Mallinckrodt Institute of Radiology, Washington University, Saint Louis, Missouri, United States of America; 5 Department of Medicine, Brigham and Women’s Hospital, Harvard Medical School, Boston, Massachusetts, United States of America; 6 Department of Obstetrics and Gynecology, Washington University, Saint Louis, Missouri, United States of America; Centre Hospitalier Universitaire Vaudois, FRANCE

## Abstract

Polyphenol-rich pomegranate juice has been shown to have benefit as a neuroprotectant in animal models of neonatal hypoxic-ischemia. No published studies have investigated maternal polyphenol administration as a potential neuroprotectant in at-risk newborns, such as those with intrauterine growth restriction (IUGR). This was a randomized, placebo-controlled, double-blind pilot study to investigate the impact of maternal pomegranate juice intake in pregnancies with IUGR, on newborn brain structure and function at term-equivalent age (TEA). Mothers with IUGR at 24–34 weeks’ gestation were recruited from Barnes-Jewish Hospital obstetrical clinic. Consented mothers were randomized to treatment (8 oz. pomegranate juice) or placebo (8 oz. polyphenol-free juice) and continued to take juice daily from enrollment until delivery (mean 20.1 and 27.1 days, respectively). Infants underwent brain MRI at TEA (36–41 weeks’ gestation). Brain measures were compared between groups including: brain injury score, brain metrics, brain volumes, diffusion tensor imaging and resting state functional connectivity. Statistical analyses were undertaken as modified intention-to-treat (including randomized participants who received their allocated intervention and whose infants received brain MRI) and per-protocol (including participants who strictly adhered to the protocol, based on metabolite status). Seventy-seven mothers were randomized to treatment (n = 40) or placebo (n = 37). Of these, 28 and 27 infants, respectively, underwent term-equivalent MRI. There were no group differences in brain injury, metrics or volumes. However, treatment subjects displayed reduced diffusivity within the anterior and posterior limbs of the internal capsule compared with placebo. Resting state functional connectivity demonstrated increased correlation and covariance within several networks in treatment subjects, with alterations most apparent in the visual network in per-protocol analyses. Direct effects on health were not found. In conclusion, maternal pomegranate juice intake in pregnancies with known IUGR was associated with altered white matter organization and functional connectivity in the infant brain, suggesting differences in brain structure and function following *in utero* pomegranate juice exposure, warranting continued investigation.

**Clinical trial registration.** NCT00788866, registered November 11, 2008, initial participant enrollment August 21, 2012.

## 1. Introduction

Hypoxic-ischemic injury is the most common contributor to brain injury in the term-born infant. Occurring in 2 per 1000 live births, it results in substantial morbidity and mortality, contributing to almost a quarter of newborn deaths worldwide [1–3]. Despite advances in neonatal intensive care, including recent widespread implementation of therapeutic hypothermia [2, 4, 5], as many as 35% of survivors experience significant long-term neurodevelopmental deficits [3, 5, 6].

Oxidative stress represents a key pathophysiological mechanism in hypoxic-ischemic neuronal injury involving free radical production and a destructive inflammatory cascade [7], with devastating consequences on the developing brain [8]. Critically, brain injury following hypoxic-ischemia is a dynamic process, evolving over hours to weeks [9]. By the time an infant with injury presents secondary degeneration has often already commenced [10, 11], limiting the potential benefit of neuroprotective strategies started *ex utero*. Shifting treatment focus to the fetus *in utero* thus represents a unique window for therapeutic intervention in hypoxic-ischemia.

Polyphenols are a promising class of neuroprotectants shown to exert their effects directly on the brain [12, 13]. These antioxidants, particularly ellagitannins, are naturally-occurring in foods such as berries, nuts, grapes and teas, and have demonstrated preventative effects in animal models of chronic disease, such as cancer, diabetes, cardiovascular, renal and neurodegenerative diseases [12, 14–18]. One of the highest polyphenol-containing dietary supplements available commercially is pomegranate juice (POM), which has been studied *in vitro* and *in vivo* without any side effects [17, 19, 20]. Animal models of hypoxic-ischemic injury have documented its neuroprotectant effects following maternal supplementation [21, 22], with evidence for dose and time-dependent effects [22]. Relatedly, polyphenols have been shown to attenuate oxidative stress and apoptosis in cultured human trophoblasts, implicating a role for p53 downregulation [23, 24]. Collectively, these studies suggest prenatal administration of polyphenols may aid in neuroprotection for infants with hypoxic-ischemic injury.

To date, no human study has investigated maternal polyphenol administration as a neuroprotectant in at-risk newborns. To test if *in utero* administration can protect against hypoxic-ischemia, a model of prenatal brain injury is required. Intrauterine growth restriction (IUGR), defined as growth *in utero* that fails to meet the endogenous potential of the fetus [25, 26], represents one such model. Importantly, fetuses with IUGR often suffer long-term placental insufficiency resulting in chronic hypoxia similar to hypoxic-ischemic injury following acute perinatal injury [27]. Furthermore, the vulnerability of the developing brain in infants with IUGR has been demonstrated in term-equivalent magnetic resonance imaging (MRI) studies [28, 29].

The current exploratory study sought to investigate relationships between maternal POM supplementation and infant brain macrostructure, microstructural organization and functional connectivity in pregnancies with IUGR.

## 2. Materials and methods

### 2.1. Trial design and participants

This was a randomized, controlled, double-blind pilot study of maternal POM consumption during pregnancy. The sample was recruited from an inner city US population, with the study conducted at Barnes-Jewish Hospital in St Louis, Missouri during 2012–2014. A study coordinator evaluated expectant mothers receiving prenatal care for enrollment. Inclusion criteria were: 1) fetal diagnosis of IUGR defined by estimated fetal weight <10^th^ percentile for gestational age [25, 26]; 2) 24–34 weeks’ gestation based on ultrasound or reliable clinical dating by ACOG standards [30]. Exclusion criteria were: 1) major congenital abnormalities; 2) known fetal chromosomal disorder; 3) maternal illicit drug use; 4) maternal HIV and/or hepatitis C infection; 4) premature rupture of membranes. All protocols were in accordance with the 1964 Helsinki declaration and its amendments or comparable ethical standards. The study was approved by the Washington University Institutional Review Board. Written informed consent was obtained for all participants.

### 2.2. Initial visit

Sociodemographic, health status and pregnancy information were collected for consented participants by questionnaires. Maternal blood was drawn at enrollment to measure baseline metabolite levels, specifically urolithin A (UA) and dimethylellagic acid glucuronide (DMEAG).

### 2.3. Randomization

Participants were randomized by a computerized random number generator to treatment or placebo in a 1:1 ratio, and completed either a daily regimen of 8 oz. of 100% pomegranate juice (POM Wonderful, Los Angeles, CA) or a control beverage, respectively. Allocation concealment was achieved using sequentially numbered opaque sealed envelopes generated using a random number generator with each opaque envelope pulled in numerical sequence. Total polyphenols in the beverages were determined by the Folin-Ciocalteu method calibrated by a gallic acid standard curve and reported as gallic acid equivalents (GAE) [20, 31, 32]. Pomegranate juice (16^o^ Brix) contained no less than 700mg GAE. The control beverage (16^o^ Brix) was polyphenol-free (no more than 38 mg GAE), matched for sensory characteristics and calorie content. The juice was labeled A or B such that the investigative team, participants and healthcare providers remained blinded. Participants were instructed to begin juice consumption following initial blood draw at enrollment through delivery.

### 2.4. Follow-up and compliance

Participants were followed from enrollment until delivery. Participants kept a daily diary documenting the number of days of juice consumption. Maternal and cord blood was collected at delivery and analyzed to determine change in polyphenol levels from baseline and confirm the presence of transferred pomegranate metabolites. Mode of delivery, complications, APGAR scores, weight, head circumference and length were recorded. Clinically-stable infants underwent term-equivalent brain MRI. While formal neurodevelopmental follow up was planned to take place at 18–24 months of age, this was not conducted due to principal investigator relocation and study cessation.

### 2.5. Image acquisition

Infants were scanned without sedation at 36–41 weeks postmenstrual age on a Siemens Trio 3T scanner (Erlangen, Germany) [33]. Images included *T*_1_-weighted (TR/TE 1550/3.05 ms, voxel size 1×1×1.3 mm^3^) and *T*_2_-weighted (TR/TE 8210/161 ms; voxel size 1×1×1 mm^3^) sequences. Diffusion data were obtained using a spin-echo echo-planar-image (EPI) sequence with 27 *b* values ranging from 0 to 1200 s/mm^2^ and spatial resolution 2×2×2 mm^3^. A gradient echo, EPI sequence sensitized to *T*_2_* blood oxygen dependent (BOLD) signal changes (TR/TE 2910/28 ms, voxel size 2.4×2.4×2.4 mm^3^) was used to collect 200 frames (~10 minutes) of resting state-functional connectivity MRI data (fcMRI). Images were interpreted by pediatric neuroradiologists (Drs. Shimony and McKinstry) and a neonatologist (TEI.).

### 2.6. Image analysis

Brain abnormalities were scored on *T*_1_- and *T*_2_-weighted images using the Kidokoro scoring system [34]. Brain metrics were calculated on aligned *T*_2_-weighted images [35]. Total and regional brain volumes were estimated using MANTiS (S1 Fig) [36]. Diffusion data was processed using a tensor model [37]. Images were registered to an in-house neonatal *T*_2_ atlas and distortion and motion corrected using FSL [38]. Regions of interest (ROIs) were manually drawn on each brain [37] using Analyze (Mayo Clinic, Rochester, MN) in the corpus callosum and bilateral anterior and posterior limbs of the internal capsule (ALIC, PLIC), optic radiations, frontal lobes, cingulum bundle and centrum semiovale, to generate diffusion tensor imaging (DTI) measures of fractional anisotropy (FA), mean (MD), radial (RD) and axial diffusivity (AD). Ten subjects were excluded from DTI analysis due to processing failure (6 control, 4 treatment).

fcMRI data was processed as previously described [39, 40]. Frames with significant motion (>0.5 mm displacement) were excluded [41]; at least 5 minutes of low-motion BOLD data was required for inclusion. Correlation matrices were constructed using 214 cortical and subcortical grey matter ROIs [42–44]. ROI:ROI correlation coefficients were computed using the Pearson product moment formula [45], and Fisher z-transformed [46]. Time series covariance estimates were computed to preserve sensitivity for detecting fcMRI alterations [45, 47]. Composite scores providing mean correlation and covariance within resting state networks (RSN) and between each network pair were computed to reduce data dimensionality and suppress sampling error [48]. Eighteen subjects were excluded from analysis due to no data (5 control, 2 treatment), motion (2 control, 8 treatment) or processing failure (1 treatment).

### 2.7. Safety assessment

Safety was assessed by analysis of adverse events; laboratory investigations, including assessment of sepsis and necrotizing enterocolitis (NEC), cardiorespiratory complications, and evaluation of intraventricular hemorrhage (IVH) on clinical ultrasound [49]. Assessors were unaware of study-group allocation.

### 2.8. Outcome measures

Primary outcomes: Term-equivalent MRI measures of brain structure and connectivity included: brain injury, metrics and volumes; ROI measures of FA, MD, AD and RD; and network composite correlation and covariance measures.

Secondary outcomes: Measures of treatment compliance included number of days of juice consumption, maternal blood metabolite concentrations (enrollment and delivery) and cord blood metabolite concentrations. Safety outcomes included NICU and special care nursery admission, and incidences of respiratory distress, resuscitation, IVH, sepsis and NEC.

### 2.9. Statistical analysis

Analyses were conducted using SAS 9.4 (SAS Institute, Cary, N.C.) and STATA 13.1 (StataCorp, Texas, USA). Power calculations were based on effect sizes with regard to the primary brain outcome measures of a comparable study in high-risk infant populations, which reported standardized effect sizes between 0.7–1.3 [50]. Accounting for the feasibility of our study, 80 subjects (40 per study arm) yields 80% power based on a two-tailed test of significance (α = 0.05) to detect effect sizes of at least 0.7.

Modified intention-to-treat (mITT) analyses were conducted including randomized participants who received their allocated intervention and who underwent brain MRI. This mITT design was necessary given the primary outcome measure–MRI measures of brain structure and connectivity–could only be assessed for infants who underwent term-equivalent brain MRI. Per-protocol (PP) analyses were conducted including participants who strictly adhered to the protocol based on metabolite status, i.e. comparing metabolite-positive (UA or DMEAG) treatment participants with metabolite-negative placebo participants. Group differences in brain outcome measures were assessed using generalized linear models adjusted for postmenstrual age at scan, except for brain injury which was assessed using Fisher’s exact test for categorical variables due to small cell numbers. Differences in compliance and safety measures were tested using *χ*^2^ or Fisher’s exact test for categorical variables and Student *t*-tests for continuous variables. For outcome variables with skewed distributions that were unable to be normalized by transformation, quantile regression was used to estimate the conditional median of the response variable. Due to the exploratory nature of this study, we did not adjust for multiple corrections [51, 52].

## 3. Results

**Fig 1:**
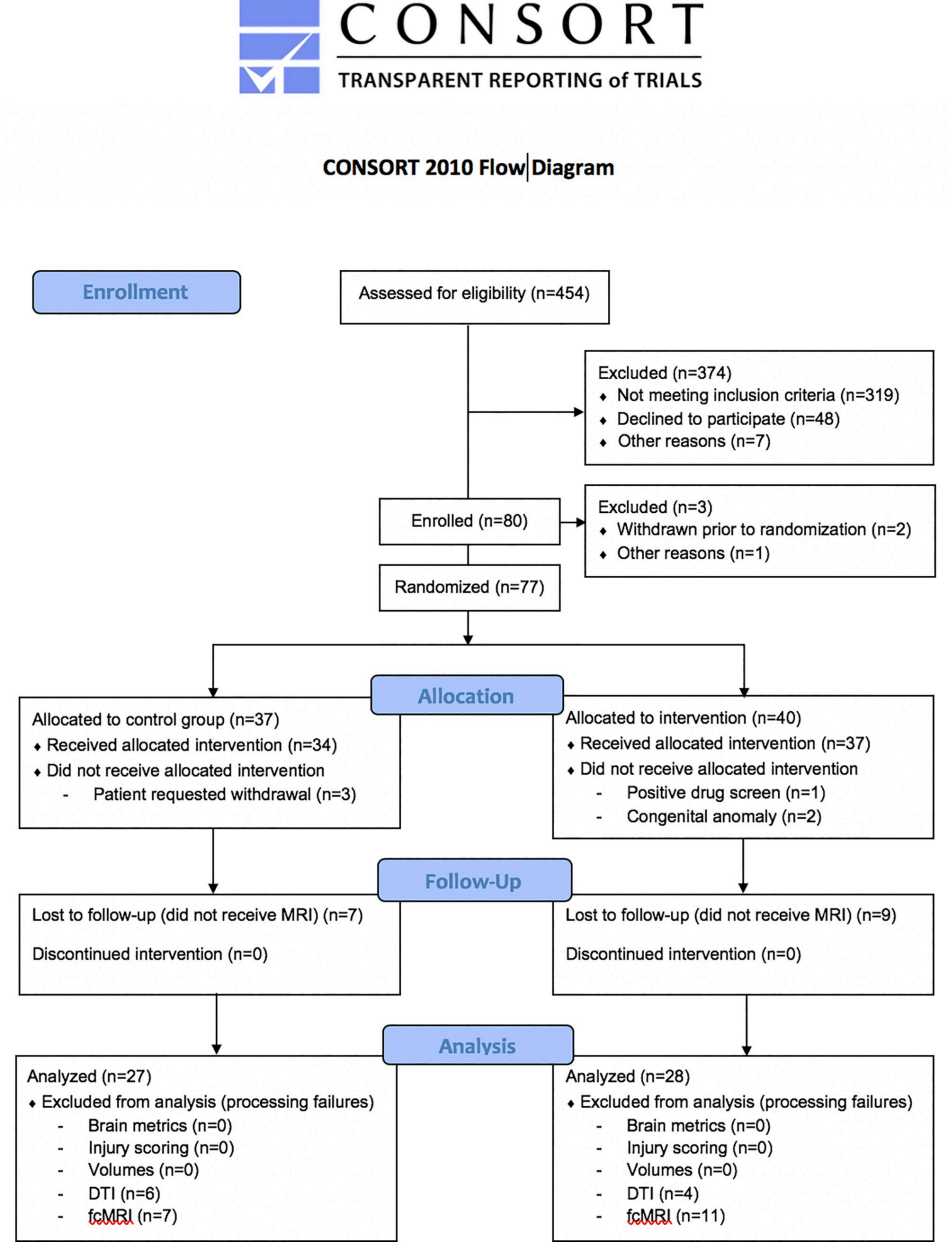
Participant flowchart.

Eighty eligible mothers were enrolled and randomly assigned to treatment and placebo groups (Fig 1). Two mothers withdrew from the study prior to randomization. One subject was excluded for technical reasons. Following randomization, 6 mothers (7.7%) did not receive the allocated intervention, of whom 3 requested withdrawal (placebo), 1 was excluded due to positive drug screen (treatment) and 2 were excluded due to congenital anomalies (treatment). A total of 34 (91.9%) placebo and 37 (90.2%) treatment subjects were followed to delivery, of whom 27 (79.4%) and 28 (75.7%) underwent term-equivalent MRI, respectively.

### 3.1. Baseline characteristics and clinical outcomes

The groups were similar in all baseline and demographic characteristics in both mITT and PP analyses, including IUGR severity as indicated by estimated fetal weight and growth percentiles at enrollment and birth weight Z-scores [53] (Table 1). There were no differences in other clinical outcomes between groups except for a modestly lower cord arterial base excess in the treatment group compared with placebo in the PP analysis. There were no differences between participants lost to follow-up (n = 22) and those who underwent infant MRI (n = 55), except for a greater percentage of preterm infants lost to follow-up (<37 weeks: 13 (59%) v. 12 (23%), p = 0.002; <34 weeks: 10 (45%) v. 3 (5%), p<0.001), although this was not associated with treatment arm.

**Table 1:** Baseline characteristics and clinical outcomes of study participants.

	**MODIFIED INTENTION-TO-TREAT**		**PER-PROTOCOL**	
**Variable**	**Placebo** **(n = 27)**	**POM** **(n = 28)**	**Placebo, Metabolite–ve** **(n = 15)**	**POM, Metabolite +ve** **(n = 17)**
*Baseline Characteristics*				
Maternal age (*years*), mean (SD)	26.9 (6.7)	24.4 (6.1)	25.9 (7.3)	24.7 (6.9)
Race, Black, *n (%)*	17 (63)	21 (75)	9 (60)	12 (71)
Race, White, *n (%)*	8 (30)	7 (25)	5 (33)	5 (29)
Smoking, *n (%)*	7 (26)	7 (25)	5 (33)	4 (24)
Substance use, *n (%)*	0 (0)	0 (0)	0 (0)	0 (0)
Sickle cell disease, *n (%)*	0 (0)	0 (0)	0 (0)	0 (0)
Gestational age at enrollment, mean (SD)	30.5 (2.6)	30.8 (2.5)	30.4 (2.8)	30.8 (2.6)
Gestational age at enrollment, median (IQR)	31 (28, 33)	31.5 (29, 32.5)	31 (28, 33)	32 (29, 33)
Estimated fetal weight at enrollment (*g*), mean (SD)	1187.3 (374.8)	1194.5 (380.0)	1205.7 (414.2)	1184.6 (428.9)
Growth percentile at enrollment, mean (SD)	6.8 (2.0)	6.0 (2.1)	7.3 (2.1)	6.5 (2.2)
Steroids for fetal lung maturity, *n (%)*	1 (4)	2 (7)	0 (0)	1 (6)
*Maternal & Delivery Outcomes*				
Gestational age at delivery, *weeks*, mean (SD)	37.4 (1.6)	36.8 (2.5)	36.9 (1.7)	37.1 (2.0)
PMA at MRI scan, *weeks*, mean (SD)	38.6 (1.3)	38.3 (1.6)	38.2 (1.2)	38.1 (1.5)
Preterm birth <37 weeks, *n (%)*	4 (15)	7 (25)	3 (20)	4 (24)
Preterm birth <34 weeks, *n (%)*	0 (0)	3 (11)	0 (0)	2 (12)
Mode of delivery (vaginal), *n (%)*	18 (67)	19 (68)	12 (80)	13 (76)
Meconium stained amniotic fluid, *n (%)*	1 (4)	1 (4)	1 (7)	1 (6)
*Neonatal Outcomes*				
Sex, *n (%)*	13 (48)	13 (46)	8 (53)	8 (47)
Birthweight (*g*), mean (SD)	2536.6 (367.5)	2385.2 (476.7)	2344.7 (289.7)	2415.9 (378.8)
Birthweight Z-score, mean (SD)	-1.03 (0.70)	-1.09 (0.55)	-1.23 (0.78)	-1.10 (0.57)
APGAR score at 1 minute, median (IQR)	8 (8, 9)	8 (8, 8)	8 (7, 8)	8 (8, 8)
APGAR score at 5 minutes, median (IQR)	9 (9, 9)	9 (8.5, 9)	9 (9, 9)	9 (9, 9)
Cord arterial pH, mean (SD)	7.29 (0.05)	7.27 (0.05)	7.30 (0.05)	7.26 (0.05)
Cord arterial base excess, mean (SD)	-2.55 (1.69)	-2.97 (1.25)	-2.54 (1.40)	-3.00 (1.34)
Respiratory distress syndrome, *n (%)*	5 (19)	6 (21)	3 (20)	1 (6)
	**MODIFIED INTENTION-TO-TREAT**		**PER-PROTOCOL**	
**Variable**	**Placebo** **(n = 27)**	**POM** **(n = 28)**	**Placebo, Metabolite–ve** **(n = 15)**	**POM, Metabolite +ve** **(n = 17)**
*Baseline Characteristics*				
Maternal age (*years*), mean (SD)	26.9 (6.7)	24.4 (6.1)	25.9 (7.3)	24.7 (6.9)
Race, Black, *n (%)*	17 (63)	21 (75)	9 (60)	12 (71)
Race, White, *n (%)*	8 (30)	7 (25)	5 (33)	5 (29)
Smoking, *n (%)*	7 (26)	7 (25)	5 (33)	4 (24)
Substance use, *n (%)*	0 (0)	0 (0)	0 (0)	0 (0)
Sickle cell disease, *n (%)*	0 (0)	0 (0)	0 (0)	0 (0)
Gestational age at enrollment, mean (SD)	30.5 (2.6)	30.8 (2.5)	30.4 (2.8)	30.8 (2.6)
Gestational age at enrollment, median (IQR)	31 (28, 33)	31.5 (29, 32.5)	31 (28, 33)	32 (29, 33)
Estimated fetal weight at enrollment (*g*), mean (SD)	1187.3 (374.8)	1194.5 (380.0)	1205.7 (414.2)	1184.6 (428.9)
Growth percentile at enrollment, mean (SD)	6.8 (2.0)	6.0 (2.1)	7.3 (2.1)	6.5 (2.2)
Steroids for fetal lung maturity, *n (%)*	1 (4)	2 (7)	0 (0)	1 (6)
*Maternal & Delivery Outcomes*				
Gestational age at delivery, *weeks*, mean (SD)	37.4 (1.6)	36.8 (2.5)	36.9 (1.7)	37.1 (2.0)
PMA at MRI scan, *weeks*, mean (SD)	38.6 (1.3)	38.3 (1.6)	38.2 (1.2)	38.1 (1.5)
Preterm birth <37 weeks, *n (%)*	4 (15)	7 (25)	3 (20)	4 (24)
Preterm birth <34 weeks, *n (%)*	0 (0)	3 (11)	0 (0)	2 (12)
Mode of delivery (vaginal), *n (%)*	18 (67)	19 (68)	12 (80)	13 (76)
Meconium stained amniotic fluid, *n (%)*	1 (4)	1 (4)	1 (7)	1 (6)
*Neonatal Outcomes*				
Sex, *n (%)*	13 (48)	13 (46)	8 (53)	8 (47)
Birthweight (*g*), mean (SD)	2536.6 (367.5)	2385.2 (476.7)	2344.7 (289.7)	2415.9 (378.8)
Birthweight Z-score, mean (SD)	-1.03 (0.70)	-1.09 (0.55)	-1.23 (0.78)	-1.10 (0.57)
APGAR score at 1 minute, median (IQR)	8 (8, 9)	8 (8, 8)	8 (7, 8)	8 (8, 8)
APGAR score at 5 minutes, median (IQR)	9 (9, 9)	9 (8.5, 9)	9 (9, 9)	9 (9, 9)
Cord arterial pH, mean (SD)	7.29 (0.05)	7.27 (0.05)	7.30 (0.05)	7.26 (0.05)
Cord arterial base excess, mean (SD)	-2.55 (1.69)	-2.97 (1.25)	-2.54 (1.40)	-3.00 (1.34)
Respiratory distress syndrome, *n (%)*	5 (19)	6 (21)	3 (20)	1 (6)

BMI–body mass index; IQR–interquartile range; POM–pomegranate; PMA–postmenstrual age; SD–standard deviation

### 3.2. Brain outcomes

**Fig 2:**
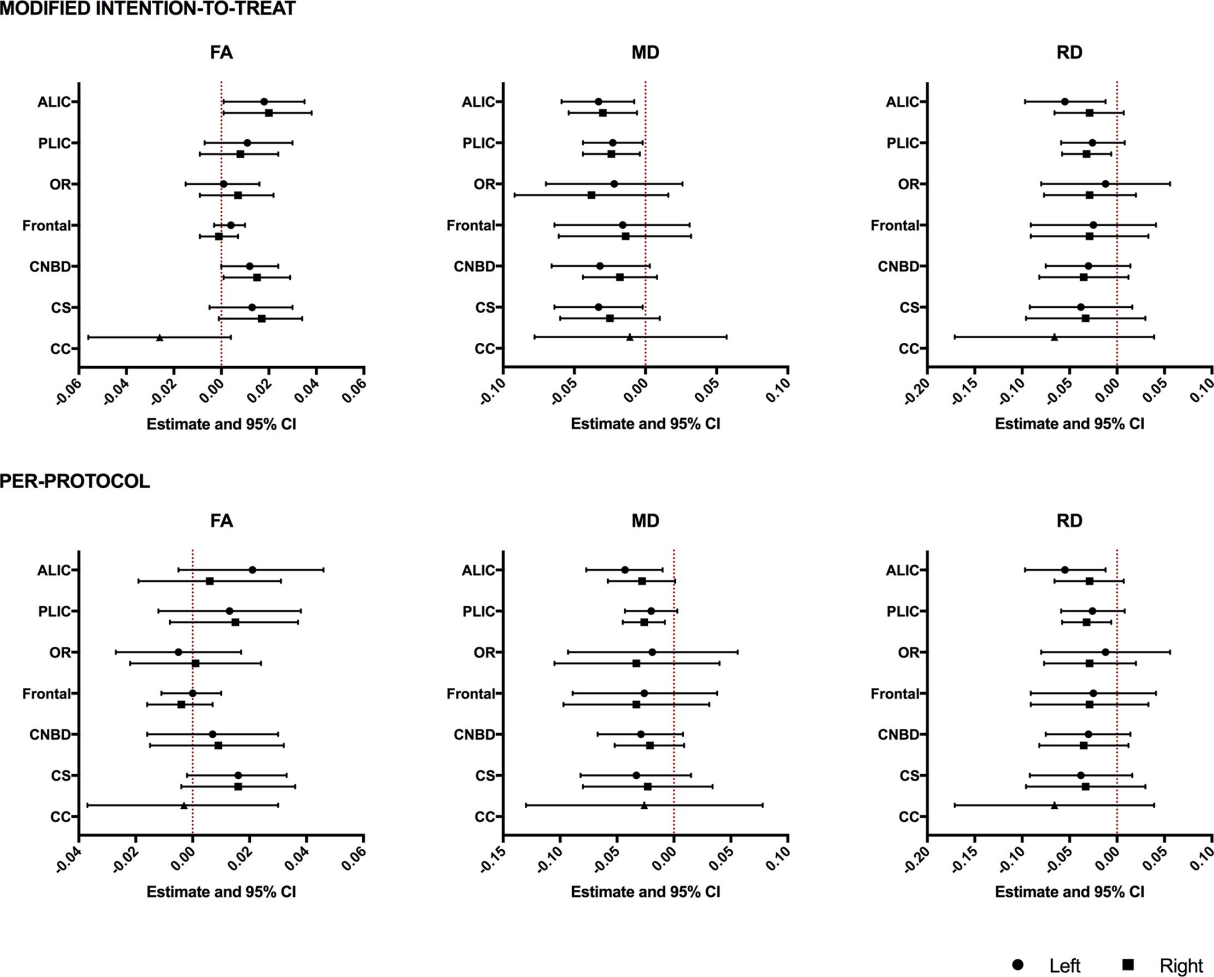
Relationships between maternal pomegranate juice intake and infant DTI measures. *(Top)* Treatment vs. placebo (modified intention-to-treat analysis). (*Bottom*) Metabolite-positive treatment vs. metabolite-negative placebo (per-protocol analysis). The point estimates represent the difference between groups in white matter DTI measures based on analyses run using generalized linear models (GLM) adjusted for postmenstrual age at scan. The error bars represent the 95% confidence intervals. ALIC–anterior limb of internal capsule; CC–corpus callosum; CNBD–cingulum bundle; CSOV–centrum semiovale; FA–fractional anisotropy; Frontal–frontal lobe; OR–optic radiation; MD–mean diffusivity; PLIC–posterior limb of internal capsule; RD–radial diffusivity. Left/Right indicate measures for bilateral white matter tracts.

While there was no difference in brain injury (S1 Table), brain metrics (S2 Table) or brain volumes (S3 Table) between treatment and placebo subjects, we observed group differences in DTI measures. Specifically, the treatment group displayed reduced MD compared with placebo in the ALIC, PLIC and centrum semiovale which was mediated by reduced RD (Fig 2A, S4 Table). These differences remained in PP analysis for the left ALIC and right PLIC (Fig 2B, S4 Table). The treatment group also demonstrated increased FA in the right ALIC and right cingulum, although these differences did not remain in PP analysis.

**Fig 3:**
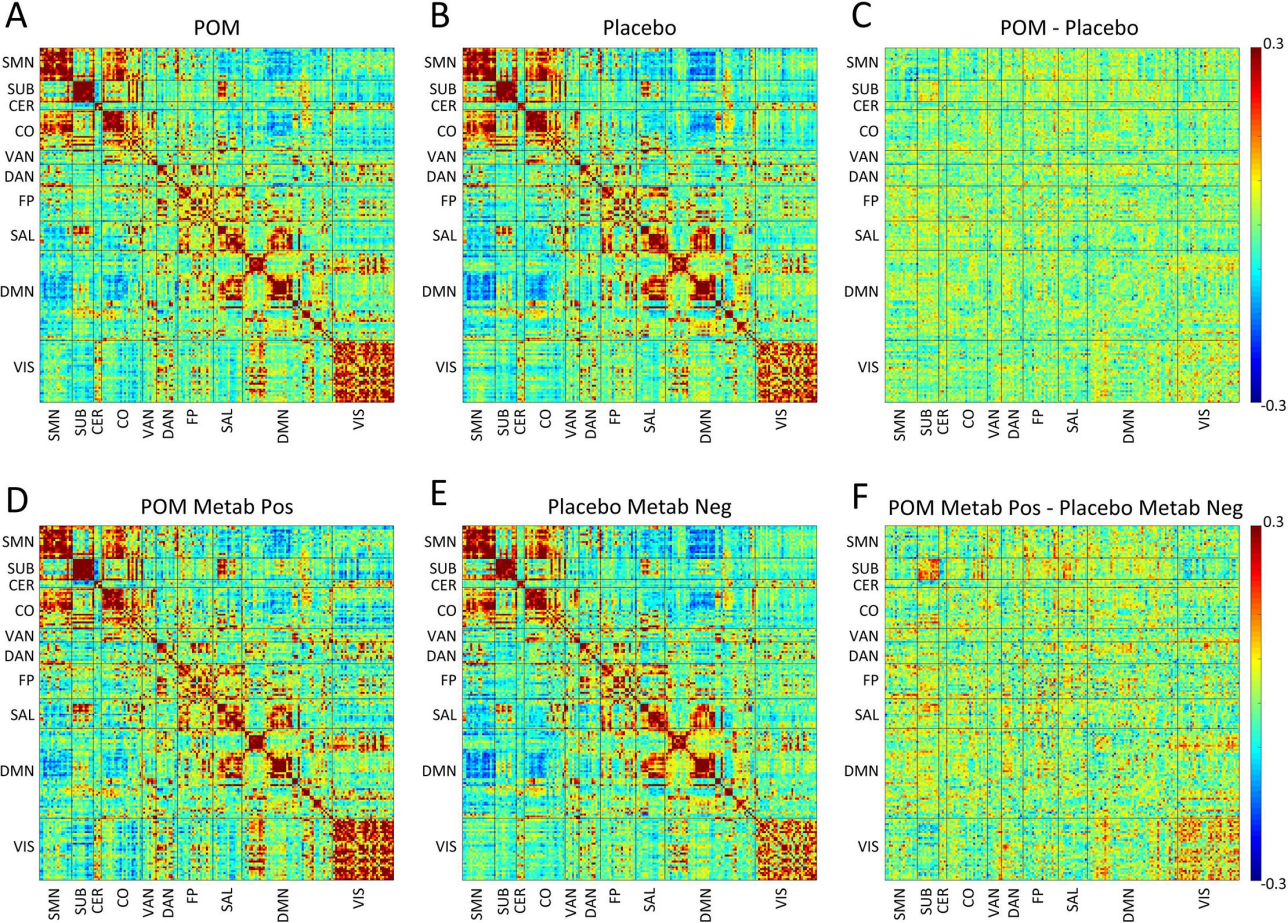
Relationships between maternal pomegranate juice intake and infant fcMRI measures (correlations). Group mean Fisher’s z-transformed correlation coefficient matrices are shown representing all ROI pairs. The block structure along the diagonal seen in both groups corresponds to resting state networks. Warm hues within diagonal blocks reflect positively correlated ROIs within RSN, while cool hues reflect negatively correlated ROIs within RSNs. (*Top*, *A-C*) Treatment vs. placebo (modified intention-to-treat analysis). (A) Infants in treatment (pomegranate juice) group and (B) infants in placebo group at term-equivalent. (C) Shows the difference between groups (treatment minus placebo). (*Bottom*, *D-F*) Metabolite-positive treatment vs. metabolite-negative placebo (per-protocol analysis). (D) Infants in metabolite-positive treatment group and (E) infants in metabolite-negative placebo group at term-equivalent. (F) Shows the difference between groups (metabolite-positive treatment minus metabolite-negative placebo). Note metabolite-positive treatment > metabolite-negative placebo correlation in subcortical and visual network. CER—cerebellar; CO—cingulo-opercular; DAN–dorsal attention network; DMN–default mode network; FP–frontal parietal network; LAN–language area network; Metab–metabolite; Neg–negative; POM–pomegranate; Pos–positive; SAL–salience network; SMN–sensorimotor network; SUB–subcortical grey matter; VAN–ventral attention network; VIS–visual network.

**Fig 4:**
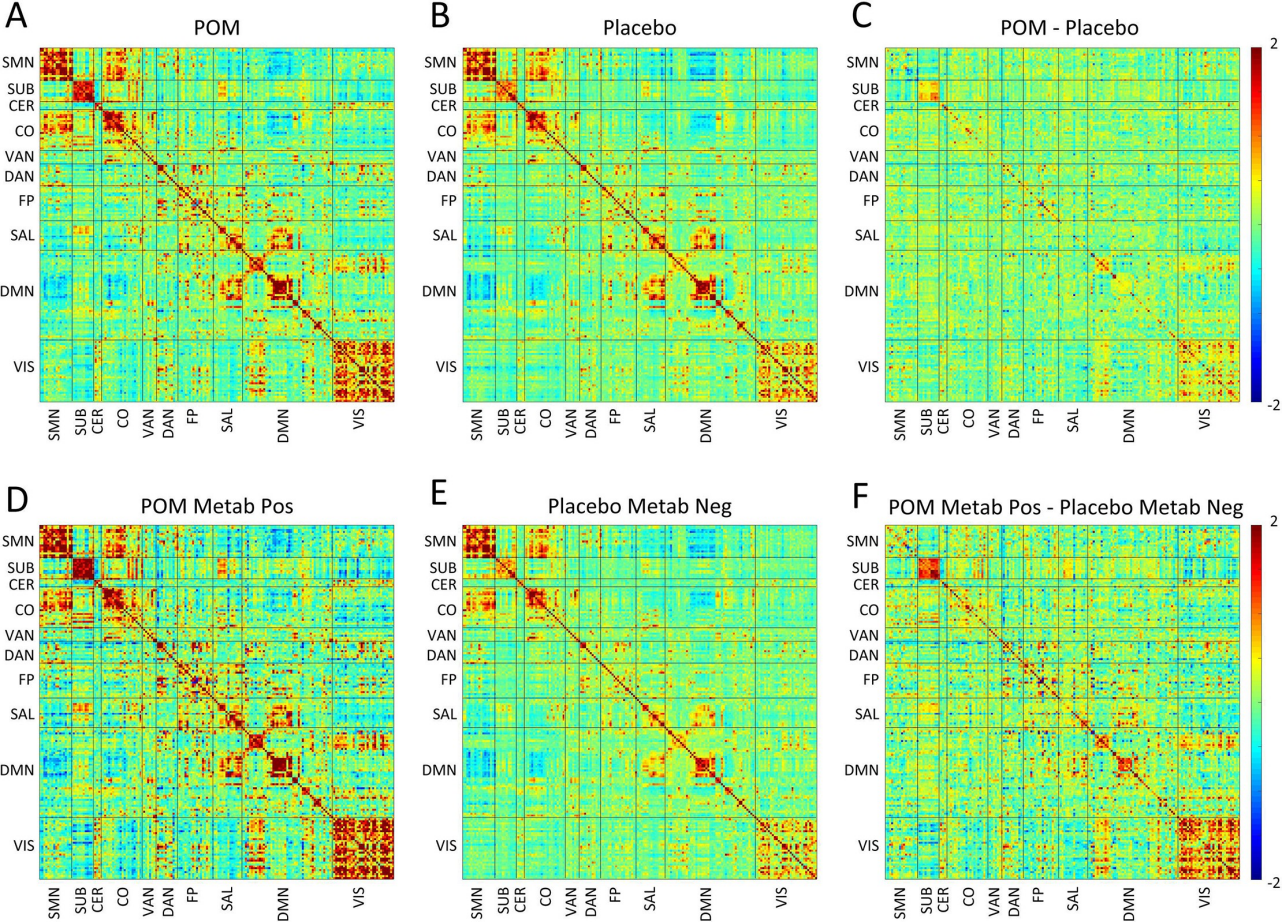
Relationships between maternal pomegranate juice intake and infant fcMRI measures (covariance). Group mean covariance matrices are shown representing all ROI pairs. The block structure along the diagonal seen in both groups corresponds to resting state networks. Warm hues within diagonal blocks reflect positively correlated ROIs within RSN, while cool hues reflect negatively correlated ROIs within RSNs. (*Top*, *A-C*) Treatment vs. placebo (modified intention-to-treat analysis). (A) Infants in treatment (pomegranate juice) group and (B) infants in placebo group at term-equivalent. (C) Shows the difference between groups (treatment minus placebo). Note treatment > placebo covariance within subcortical and visual networks. (*Bottom*, *D-F*) Metabolite-positive treatment vs. metabolite-negative placebo (per-protocol analysis). (D) Infants in metabolite-positive treatment group and (E) infants in metabolite-negative placebo group at term-equivalent. (F) Shows the difference between groups (metabolite-positive treatment minus metabolite-negative placebo). Note metabolite-positive treatment > metabolite-negative placebo covariance within subcortical and visual networks. CER—cerebellar; CO—cingulo-opercular; DAN–dorsal attention network; DMN–default mode network; FP–frontal parietal network; LAN–language area network; Metab–metabolite; Neg–negative; POM–pomegranate; Pos–positive; SAL–salience network; SMN–sensorimotor network; SUB–subcortical grey matter; VAN–ventral attention network; VIS–visual network.

fcMRI correlation and covariance matrices from mITT and PP analyses are shown in Figs 3 and 4. RSNs demonstrating the greatest differences in the treatment group are identifiable in Table 2, S2 and S3 Figs, which include composite mean correlation and covariance measures. In the mITT analysis, there was increased correlation in the subcortical network in treatment compared with placebo subjects (Table 2, Fig 3, S2 Fig). Further, in PP analysis we observed increased fcMRI measures in several networks in metabolite-positive treatment subjects, relative to metabolite-negative controls. Specifically, there was increased correlation within the visual network (Table 2, Fig 3, S2 Fig) and increased covariance within subcortical, visual, salience and default mode networks (Table 2, Fig 4, S3 Fig).

**Table 2:** Composite mean Fisher z-transformed correlations and mean covariance by group status, adjusted for postmenstrual age at scan.

	Group Summaries								Group Comparisons^1^							
	POM (n = 17)		POM, Metabolite +ve (n = 10)		Placebo (n = 20)		Placebo, Metabolite -ve (n = 14)		MODIFIED INTENTION-TO-TREAT				PER-PROTOCOL			
**Network correlation**	**Mean**	**SD**	**Mean**	**SD**	**Mean**	**SD**	**Mean**	**SD**	**Estimate**	**SE**	**t**	**P**	**Estimate**	**SE**	**t**	**P**
SMN	0.26	0.06	0.26	0.07	0.28	0.05	0.28	0.06	-0.02	0.02	-1.04	0.30	-0.02	0.03	-0.57	0.57
SUB^2^	0.41		0.42		0.34		0.33		0.09	0.04	2.23	0.03	0.11	0.08	1.45	0.15
CER	0.25	0.14	0.22	0.11	0.22	0.10	0.22	0.10	0.04	0.04	0.89	0.38	0.00	0.04	-0.05	0.96
CO^2^	0.18		0.18		0.17		0.15		-0.005	0.02	-0.21	0.84	0.02	0.03	0.71	0.48
VAN	0.13	0.04	0.14	0.03	0.12	0.05	0.11	0.05	0.01	0.02	0.89	0.38	0.03	0.02	1.47	0.16
DAN	0.12	0.06	0.12	0.07	0.11	0.05	0.12	0.05	0.00	0.02	0.04	0.97	0.00	0.02	0.14	0.89
FP	0.14	0.05	0.15	0.06	0.12	0.04	0.13	0.04	0.01	0.01	0.68	0.50	0.02	0.02	0.86	0.40
SAL	0.17	0.05	0.19	0.04	0.18	0.07	0.16	0.06	-0.01	0.02	-0.57	0.57	0.02	0.02	1.04	0.31
DMN	0.08	0.02	0.08	0.03	0.07	0.02	0.07	0.02	0.00	0.01	0.20	0.84	0.00	0.01	0.34	0.74
VIS	0.22	0.08	0.25	0.07	0.19	0.08	0.16	0.07	0.04	0.03	1.34	0.19	0.09	0.03	2.87	0.01
**Network covariance**	**Mean**	**SD**	**Mean**	**SD**	**Mean**	**SD**	**Mean**	**SD**	**Estimate**	**SE**	**t**	**P**	**Estimate**	**SE**	**t**	**P**
SMN^2^	1.30		1.51		1.33		1.28		-0.09	0.15	-0.61	0.55	0.12	0.19	0.61	0.55
SUB^2^	0.97		1.05		0.84		0.80		0.29	0.24	1.21	0.24	0.72	0.30	2.40	0.03
CER^2^	0.85		0.86		0.55		0.55		0.15	0.10	1.41	0.17	0.13	0.13	1.01	0.32
CO^2^	0.70		0.81		0.47		0.43		0.15	0.19	0.78	0.44	0.44	0.23	1.92	0.07
VAN^3^	0.38		0.50		0.26		0.24		0.10	0.07	1.48	0.15	0.26	0.13	2.01	0.06
DAN^2^	0.34		0.47		0.30		0.27		0.20	0.24	0.83	0.41	0.46	0.31	1.46	0.16
FP^2^	0.45		0.50		0.30		0.28		0.14	0.09	1.57	0.13	0.20	0.13	1.48	0.15
SAL^2^	0.43		0.62		0.35		0.26		0.10	0.24	0.40	0.69	0.68	0.23	2.97	0.01
DMN^2^	0.20		0.32		0.20		0.17		0.21	0.18	1.18	0.25	0.55	0.21	2.61	0.02
VIS^2^	0.73		1.09		0.45		0.33		0.41	0.26	1.58	0.12	0.99	0.32	3.10	0.01

CER—cerebellar; CO—cingulo-opercular; DAN–dorsal attention network; DMN–default mode network; FP–frontal parietal network; LAN–language area network; POM–pomegranate; SAL–salience network; SMN–sensorimotor network; SUB–subcortical grey matter; VAN–ventral attention network; VIS–visual network

^1^ Analyses run using generalized linear models (GLM) adjusted for postmenstrual age at scan

^2^ Distribution skewed (> |0.8|). Analyses run using ln transformed variables (for CER_avg_covariance tranformation based on raw value + 1 to account for negative values). Group summary values reflect medians, not means, of the raw distribution

^3^ Remained skewed after transformation. Raw variable modeled using quantile (median) regression (SAS PROC QUANTREG); group summary values reflect medians, not means, of the distribution

### 3.3. Compliance

Juice consumption and maternal metabolite presence (either UA or DMEAG present) at enrollment did not differ between groups (Table 3). There was a higher incidence of maternal metabolite presence at delivery in the treatment group than placebo, however, almost one-third of treatment subjects did not have maternal metabolites and almost one-half of placebo subjects had metabolites. Sixty-one percent of treatment and 33% of placebo subjects tested positive for cord blood metabolite presence. To address this variability and allow stricter investigation of relationships between POM intake and brain structure and function, PP analyses were performed using only metabolite-positive treatment, and metabolite-negative placebo subjects (Table 3).

**Table 3:** Measures of compliance.

	MODIFIED INTENTION-TO-TREAT							PER-PROTOCOL						
	Placebo (n = 27)	POM (n = 28)	Absolute Effect Size^1^	Relative Effect Size^2^	SE^2^	*t/χ*^2^	P^3^	Placebo, Metabolite -ve (n = 15)	POM, Metabolite +ve (n = 17)	Absolute Effect Size^1^	Relative Effect Size^2^	SE^2^	*t/χ*^2^	P^3^
Days of consumption, median (IQR)	23 (14, 40)	18.5 (7, 30.5)	6.8	0.44	4.17	1.6	0.11	23 (15, 40)	18 (7, 24)	9.7	0.71	4.89	2.0	0.06
Maternal UA or DMEAG at enrollment, *n (%)*	3 (11)	2 (8)^a^	-3.1%	0.64	0.08		1.00	1 (7)	1 (7)	0.0%	1.00	0.14		1.00
Maternal UA or DMEAG at delivery, *n (%)*	11 (42)^b^	17 (68)^c^	25.7%	1.49	0.13	3.4	0.07	0 (0)	17 (100)	100.0%	31.11	0.17		<0.001
Cord UA or DMEAG at delivery, *n (%)*	8 (33)^d^	14 (61)^e^	27.5%	1.69	0.13	3.6	0.06	0 (0)	14 (93)	93.3%	25.78	0.17		<0.001
Positive for metabolites in cord blood or maternal blood at delivery	11 (42)^f^	17 (68)^g^	25.7%	1.49	0.13	3.4	0.07	0 (0)	17 (100)	100.0%	31.11	0.17		<0.001

DMEAG; dimethylellagic acid glucuronid; IQR–interquartile range; POM–pomegranate; SE–standard error; UA–urolithin A

^1^ Absolute effect size calculated as the mean difference for continuous variables, and the risk difference (%) for categorical variables (Placebo = reference)

^2^ Relative effect size calculated as Cohen’s *d* for continuous variables, and the relative risk for categorical variables (Placebo = reference). For analyses with zero in one or more cells, 0.5 was added to each cell prior to calculation of the relative risk and its standard error (SE). Corresponding SE are reported.

^3^ Fisher’s exact test (2-sided) used to compare proportions by group where expected cell size < 5.

^a^ n = 25

^b^ n = 26

^c^ n = 25

^d^ n = 24

^e^ n = 23

^f^ n = 26

^g^ n = 25

### 3.4. Safety assessment

There were no group differences with respect to any complications (Table 4). Specifically, there were no complications noted in either group consistent with pulmonary hypertension or any clinical evidence consistent with premature closure of a patent ductus arteriosus (PDA). As no adverse events were found to be attributable to POM, no stopping rules were implemented.

**Table 4:** Safety assessment.

Complication	Placebo (n = 34)	POM (n = 37)	Risk Difference^1^ (%)	Relative Risk^2^	SE^2^	*χ*^2^	P^3^
NICU admission, *n (%)*	1 (3)	3 (8)	5.1	2.76	0.05		0.62
Special care nursery admission, *n (%)*	10 (29)	10 (27)	-2.4	0.92	0.11	0.05	0.82
Respiratory distress, *n (%)*	7 (21)	9 (24)	3.7	1.18	0.10	0.14	0.71
Resuscitation at delivery, *n (%)*	5 (15)	8 (22)	6.9	1.47	0.09	0.57	0.45
Intraventricular hemorrhage, *n (%)*	0 (0)	0 (0)	0.0	-	-	NA	NA
Sepsis, *n (%)*	2 (0)	1 (3)	-3.2	0.46	0.05		1.00
Necrotizing enterocolitis, *n (%)*	0 (0)	0 (0)	0	-	-	NA	NA

NICU–neonatal intensive care; POM–pomegranate; SE–standard error

^1^ Absolute effect size calculated as the mean difference for continuous variables, and the risk difference (%) for categorical variables (Placebo = reference)

^2^ Relative effect size calculated as Cohen’s *d* for continuous variables, and the relative risk for categorical variables (Placebo = reference). For analyses with zero in one or more cells, 0.5 was added to each cell prior to calculation of the relative risk and its standard error (SE). Corresponding SE are reported.

^3^ Fisher’s exact test (2-sided) used to compare proportions by group where expected cell size < 5.

## 4. Discussion

This is the first study reporting relationships between maternal POM intake and brain structure and function in infants with IUGR. While we did not observe differences in brain macrostructure, we report regional differences in white matter microstructure and functional connectivity in association with POM intake, suggesting potential *in utero* effects on the newborn brain.

The antioxidant effects of POM have been demonstrated *in vitro* and *in vivo* without any proven side effects [17, 19, 20]. Loren *et al*. reported over 60% reduction in brain tissue loss in mice with hypoxic-ischemia born to dams receiving POM [21]. Further, maternal POM supplementation was recently shown to attenuate maternal inflammation-induced rat fetal brain injury [54], and rescue hypoxia-induced fetal growth restriction in pregnant mice [55]. In the current study, the lack of an observable association with brain injury may reflect its low incidence, which necessitated grouping all infants with injury and possibly masked potential benefits in those with milder injury. Indeed, therapeutic hypothermia has shown greater benefits in subjects with less severe injury [56].

Brain metrics have been shown to reliably quantify growth in high-risk infants, such as those born preterm [35, 57]. However, they are only a surrogate measure of brain volume, with limited sensitivity for detecting group differences in small samples. Volumetric analyses have demonstrated regional vulnerabilities within the hippocampus, brainstem and thalamus, and the watershed zone in term newborns with hypoxic-ischemic injury [58, 59]. The existence of corresponding regional neuroprotective effects of polyphenols remains unclear. While we were likely underpowered to detect such effects, animal studies have reported protective effects in hippocampal and cortical regions [21, 60–69].

It is plausible that responses to POM are partially mediated via mechanisms below the macrostructural level. DTI investigations offer a complementary approach to conventional MRI, providing added insight regarding microscopic white matter organization. Here, we observed regional microstructural alterations in association with maternal POM intake, implicating the ALIC and PLIC, cingulum and centrum semiovale; areas previously shown to be vulnerable to the effects of hypoxic-ischemia [58, 70]. These findings were present in both mITT and PP analyses. While definitive interpretation of DTI changes remains complicated [71, 72], increased FA is observed in more mature white matter [73], while reduced FA likely reflects axonal damage, reduced myelination and/or reduced neuroglial packing density [74, 75], and is often reported in very preterm infants (VPT) relative to term born peers [76]. MD is a summary measure of diffusivity, with increased values reflecting immature, poorly developed white matter [75, 77], as often seen in VPT infants compared with full term peers [76]. AD and RD provide complementary information; the former reflecting axonal organization and density, with RD reflecting alterations in myelination [75, 78]. Thus, we postulate *in utero* POM exposure may lead to improved maturation, via neuroprotective mechanisms such as axonal sparing and/or remyelination. This appears to be supported by evidence of improved motor function in association with pomegranate supplementation in mice models of cytotoxic radiation [79], and cognitive and behavioral improvements in mouse models of Alzheimer’s [80], implicating a role for enhanced synaptic plasticity [81].

*In utero* POM exposure may also lead to changes in functional networks in the developing brain. fcMRI delineates the brain’s functional architecture, including widely-distributed, cortically-based RSNs known to be co-activated by task [82], several of which have been described in infants [39, 47]. In the current study, metabolite-positive treatment subjects displayed increased connectivity, characterized by greater correlation and covariance, within visual, subcortical, salience and default mode networks. Consistent with previous reports, covariance was more sensitive to these alterations than correlation measures [45]. Of note, the most apparent alterations were observed in the visual cortex, an early maturing, developmentally vulnerable cortical region [83, 84]. Interestingly, these findings survived supplemental analyses correcting for multiple comparisons using the false discovery rate [85]. The retina has been increasingly shown to be susceptible to the effects of hypoxic-ischemia [86, 87], with mechanisms involving glutamate excitotoxicity and oxidative glutamate toxicity implicated [88, 89]. Furthermore, studies have demonstrated the therapeutic potential of polyphenols in reducing oxidative stress-induced death of retinal ganglion and pigment epithelial cells [90–93].

Of note, there was little evidence for microstructural changes within corresponding white matter tracts such as the optic radiations. While some studies demonstrate robust relationships between DTI and fcMRI findings [94, 95], others have not [96–98]^,^, suggesting that structural and functional connectivity may not interrelate via a one-to-one relationship. Future studies combining these modalities are likely to improve our understanding of structure-function interactions in the developing brain. It is worth noting that the observed microstructural and functional connectivity alterations may be related to later neurodevelopmental outcomes; investigations are currently underway to better understand these relationships. Further, though the current study employed term-equivalent MRI, investigations including fetal MRI may provide additional insight into brain development trajectories following *in utero* POM exposure.

The current study supports the therapeutic potential of polyphenols such as POM for hypoxic-ischemic injury, however, our findings need to be considered with respect to several limitations. In the current study consumption of 8 oz. POM provided a daily administration of no less than 700 mg GAE of polyphenols [20]. Ellagitannins are metabolized by colonic gut microbiota to generate more readily absorbed metabolites, specifically UA and DMEAG [99–101], proposed to be the bioactive molecules underlying the neuroprotectant effects of POM [99–102]. However, there are challenges associated with the chemical estimation methods employed to assess metabolite bioavailability [60], further compounded by intra-subject variability in polyphenol metabolism arising from genetic polymorphisms in metabolizing enzymes [100] as well as variations in gut microbiota [103]. There is also limited information on, and variability in, polyphenol content in various food items [60, 104, 105]. While we did not capture information on participant diet, it is reasonable to assume that residual polyphenol-intake from non-pomegranate dietary sources was randomly distributed among study participants given UA and DMEAG were largely absent in both groups at enrolment. Nonetheless, in PP analyses using metabolite positivity as a proxy for polyphenol intake, confounding arising from these factors cannot be excluded. Finally, we did not undertake echocardiography for evaluation of the PDA but there was no clinical evidence seen of premature closure or constriction of the PDA. Direct effects on health were not found.

## 5. Conclusion

We provide preliminary evidence supporting potential *in utero* effects of POM exposure. Specifically, we report differences suggestive of enhanced microstructural organization within the ALIC and PLIC and functional connectivity within the visual network. This study provides a foundation for building an evidence base for potential neuroprotective agents that mat be effective in the general population of at-risk newborns, such as those with hypoxic-ischemic injury. Importantly, our findings warrant the need for a larger, rigorously designed clinical trial to allow continued investigation into the potential region-specific neuroprotective effects of polyphenols.

## Supporting information

S1 Checklist CONSORT checklist. (DOC)

S1 Fig Brain volumetry using MANTiS segmentation in a representative subject. Red = cortical grey matter; green = white matter; yellow = deep grey matter; grey = cerebellum; purple = brainstem; blue = cerebrospinal fluid; cyan = amygdala.
(PDF)

S2 Fig Relationships between maternal pomegranate juice intake and infant fcMRI measures (network average correlations). Composite mean Fisher’s z-transformed correlation matrices are shown representing averages over ROI pairs within each network (*Top*, *A-C*) Treatment vs. placebo (intention-to-treat analysis). (A) Infants in treatment (pomegranate juice) group and (B) infants in placebo group at term-equivalent. (C) Shows the difference between groups (treatment minus placebo). (*Bottom*, *D-F*) Metabolite-positive treatment vs. metabolite-negative placebo (per-protocol analysis). (D) Infants in metabolite-positive treatment group and (E) infants in metabolite-negative placebo group at term-equivalent. (F) Shows the difference between groups (metabolite-positive treatment minus metabolite-negative placebo). Note metabolite-positive treatment > metabolite-negative placebo network average correlation in subcortical and visual network. CER—cerebellar; CO—cingulo-opercular; DAN–dorsal attention network; DMN–default mode network; FP–frontal parietal network; LAN–language area network; Metab–metabolite; Neg–negative; POM–pomegranate; Pos–positive; SAL–salience network; SMN–sensorimotor network; SUB–subcortical grey matter; VAN–ventral attention network; VIS–visual network.
(PDF)

S3 Fig Relationships between maternal pomegranate juice intake and infant fcMRI measures (network average covariance). Composite mean covariance matrices are shown representing averages over ROI pairs within each network (*Top*, *A-C*) Treatment vs. placebo (modified intention-to-treat analysis). (A) Infants in treatment (pomegranate juice) group and (B) infants in placebo group at term-equivalent. (C) Shows the difference between groups (treatment minus placebo). (*Bottom*, *D-F*) Metabolite-positive treatment vs. metabolite-negative placebo (per-protocol analysis). (D) Infants in metabolite-positive treatment group and (E) infants in metabolite-negative placebo group at term-equivalent. (F) Shows the difference between groups (metabolite-positive treatment minus metabolite-negative placebo). Note metabolite-positive treatment > metabolite-negative placebo network average covariance within subcortical and visual networks. CER—cerebellar; CO—cingulo-opercular; DAN–dorsal attention network; DMN–default mode network; FP–frontal parietal network; LAN–language area network; Metab–metabolite; Neg–negative; POM–pomegranate; Pos–positive; SAL–salience network; SMN–sensorimotor network; SUB–subcortical grey matter; VAN–ventral attention network; VIS–visual network.
(PDF)

S1 Table Brain injury by group status, adjusted for postmenstrual age at scan. (DOCX)

S2 Table Brain metrics by group status, adjusted for postmenstrual age at scan. (DOCX)

S3 Table Brain volumes by group status, adjusted for postmenstrual age at scan. (DOCX)

S4 Table DTI measures by group status, adjusted for postmenstrual age at scan. (DOCX)

S1 File Study protocol. (DOC)
