# Supplementary figures and images for: Maternal pomegranate juice intake and brain structure and function in infants with intrauterine growth restriction: A randomized controlled pilot study
Source: PLoS One. 2019 Aug 21;14(8):e0219596. doi: 10.1371/journal.pone.0219596 (PMC6703683; doi:10.1371/journal.pone.0219596)

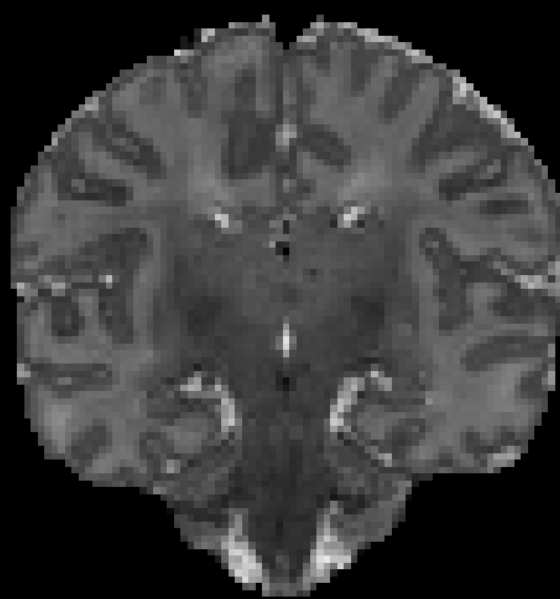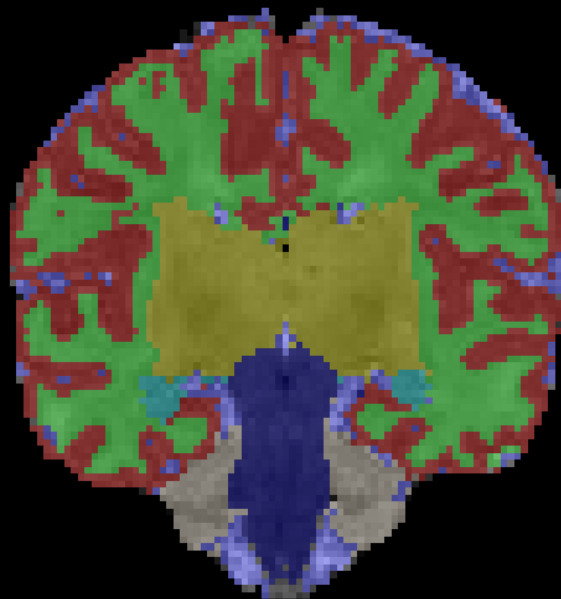

Supplement: S1 Fig — Red = cortical grey matter; green = white matter; yellow = deep grey matter; grey = cerebellum; purple = brainstem; blue = cerebrospinal fluid; cyan = amygdala. (PDF) [file pone.0219596.s002.pdf]
