# Supplementary material for: Maternal pomegranate juice intake and brain structure and function in infants with intrauterine growth restriction: A randomized controlled pilot study
Source: PLoS One. 2019 Aug 21;14(8):e0219596. doi: 10.1371/journal.pone.0219596 (PMC6703683; doi:10.1371/journal.pone.0219596)
Supplement: S3 Fig — Composite mean covariance matrices are shown representing averages over ROI pairs within each network (Top, A-C) Treatment vs. placebo (modified intention-to-treat analysis). (A) Infants in treatment (pomegranate juice) group and (B) infants in placebo group at term-equivalent. (C) Shows the difference between groups (treatment minus placebo). (Bottom, D-F) Metabolite-positive treatment vs. metabolite-negative placebo (per-protocol analysis). (D) Infants in metabolite-positive treatment group and (E) infants in metabolite-negative placebo group at term-equivalent. (F) Shows the difference between groups (metabolite-positive treatment minus metabolite-negative placebo). Note metabolite-positive treatment > metabolite-negative placebo network average covariance within subcortical and visual networks. CER—cerebellar; CO—cingulo-opercular; DAN–dorsal attention network; DMN–default mode network; FP–frontal parietal network; LAN–language area network; Metab–metabolite; Neg–negative; POM–pomegranate; Pos–positive; SAL–salience network; SMN–sensorimotor network; SUB–subcortical grey matter; VAN–ventral attention network; VIS–visual network. (PDF) [file pone.0219596.s004.pdf]

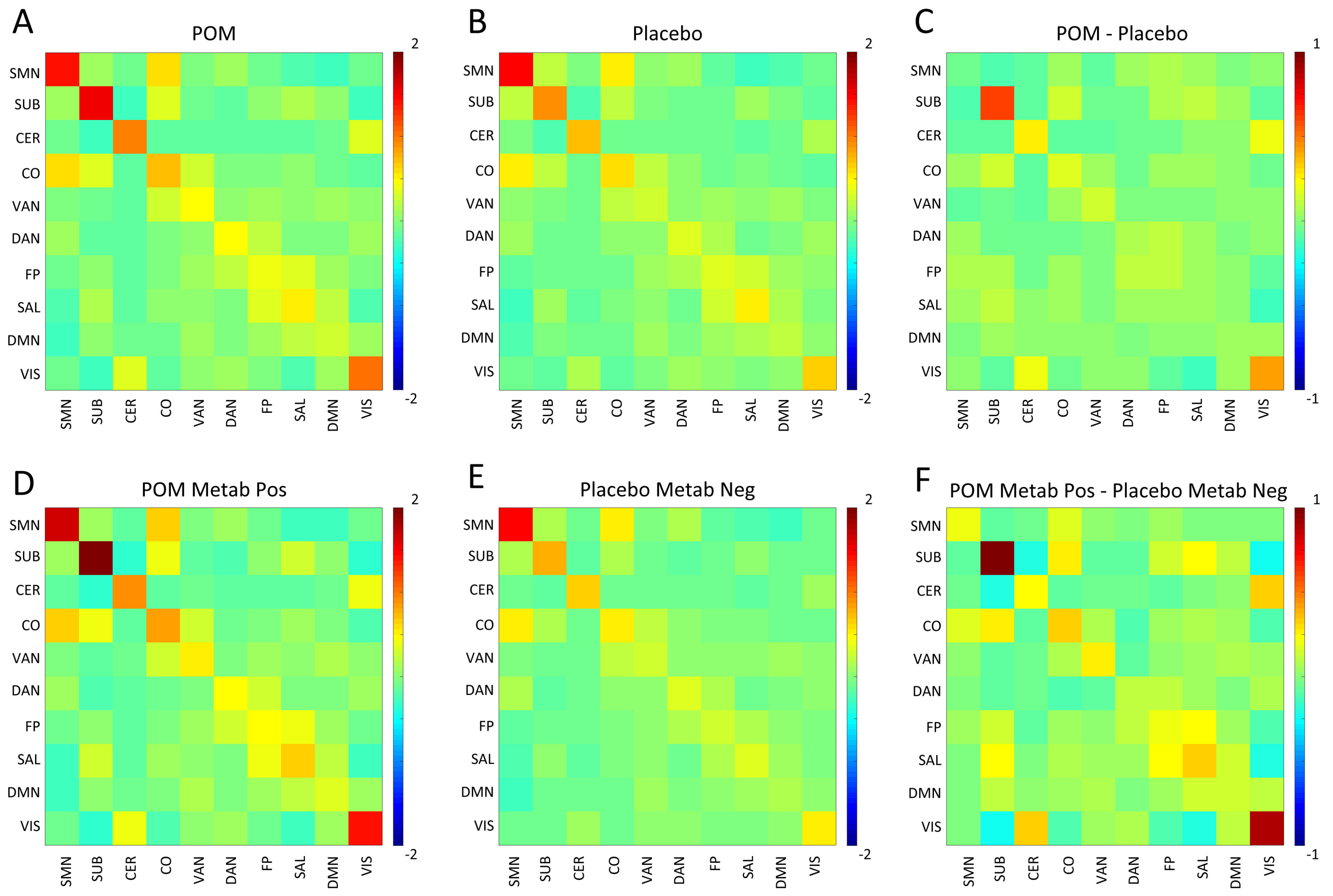
A
POM
B
Placebo
C
POM - Placebo
D
POM Metab Pos
E
Placebo Metab Neg
F
POM Metab Pos - Placebo Metab Neg
SMN
SUB
CER
CO
VAN
DAN
FP
SAL
DMN
VIS
2
-2
1
-1

S2 Figure. Relationships between maternal pomegranate juice intake and infant fcMRI measures (network average covariance). Composite mean covariance matrices are shown representing averages over ROI pairs within each network (*Top, A-C*) Treatment vs. placebo (modified intention-to-treat analysis). (A) Infants in treatment (pomegranate juice) group and (B) infants in placebo group at term-equivalent. (C) Shows the difference between groups (treatment minus placebo). (*Bottom, D-F*) Metabolite-positive treatment vs. metabolite-negative placebo (per-protocol analysis). (D) Infants in metabolite-positive treatment group and (E) infants in metabolite-negative placebo group at term-equivalent. (F) Shows the difference between groups (metabolite-positive treatment minus metabolite-negative placebo). Note metabolite-positive treatment > metabolite-negative placebo network average covariance within subcortical and visual networks. CER - cerebellar; CO - cingulo-opercular; DAN – dorsal attention network; DMN – default mode network; FP – frontal parietal network; LAN – language area network; Metab – metabolite; Neg – negative; POM – pomegranate; Pos – positive; SAL – salience network; SMN – sensorimotor network; SUB – subcortical grey matter; VAN – ventral attention network; VIS – visual network.
