# Supplementary material for: Maternal pomegranate juice intake and brain structure and function in infants with intrauterine growth restriction: A randomized controlled pilot study
Source: PLoS One. 2019 Aug 21;14(8):e0219596. doi: 10.1371/journal.pone.0219596 (PMC6703683; doi:10.1371/journal.pone.0219596)
Supplement: S1 Table — (DOCX) [file pone.0219596.s005.docx]

**S1 Table.** Brain injury by group status, adjusted for postmenstrual age at scan

|  | Group Summaries | | | | | | | | Group Comparisons | | | | | | | |
| --- | --- | --- | --- | --- | --- | --- | --- | --- | --- | --- | --- | --- | --- | --- | --- | --- |
|  | **POM**  **(n=28)** | | **POM,  Metabolite +ve**  **(n=17)** | | **Placebo**  **(n=27)** | | **Placebo,  Metabolite –ve**  **(n=15)** | | **MODIFIED INTENTION-TO-TREAT** | | | | **PER-PROTOCOL** | | | |
|  | **Grade  0** | **Grade 1-2** | **Grade  0** | **Grade 1-2** | **Grade  0** | **Grade 1-2** | **Grade  0** | **Grade 1-2** | **RD^1^ (%)** | **RR^2^** | **SE^2^** | **P^3^** | **RD^1^ (%)** | **RR^2^** | **SE^2^** | **P^3^** |
| WM cystic lesion | 27 (96.4) | 1 (3.6) | 17 (100) | 0 (0) | 27 (100) | 0 (0) | 15 (100) | 0 (0) | 3.6 | 2.90 | 0.05 | 1.00 | 0.0 | - | - | NA |
| WM signal abnormality | 24 (85.7) | 4 (14.3) | 14 (82.4) | 3 (17.7) | 19 (70.4) | 8 (13.8) | 10 (66.7) | 5 (33.3) | -15.3 | 0.48 | 0.11 | 0.21 | -15.7 | 0.53 | 0.15 | 0.42 |
| WM myelination delay | 26 (92.9) | 2 (7.1) | 16 (94.1) | 1 (5.9) | 26 (96.3) | 1 (3.7) | 15 (100) | 0 (0) | 3.4 | 1.93 | 0.06 | 1.00 | 5.9 | 2.67 | 0.08 | 1.00 |
| GM signal abnormality | 27 (96.4) | 1 (3.6) | 16 (94.1) | 1 (5.9) | 26 (96.3) | 1 (3.7) | 15 (100) | 0 (0) | -0.1 | 0.96 | 0.05 | NA | 5.9 | 2.67 | 0.08 | NA |
| DGM signal abnormality | 28 (100) | 0 (0) | 17 (100) | 0 (0) | 27 (100) | 0 (0) | 14 (93.3) | 1 (6.7) | 0.0 | - | - | 0.24 | -6.7 | 0.30 | 0.08 | 0.47 |
| CER signal abnormality | 28 (100) | 0 (0) | 17 (100) | 0 (0) | 25 (92.6) | 2 (7.4) | 15 (100) | 0 (0) | 0.0 | - | - | 1.00 | 0.0 | - | - | 1.00 |
| IVH grade abnormality | 23 (82.1) | 5 (17.9) | 14 (82.4) | 3 (17.7) | 23 (85.2) | 4 (14.8) | 12 (80) | 3 (16.7) | 3.0 | 1.21 | 0.10 | 1.00 | -2.3 | 0.88 | 0.14 | 1.00 |

Group summaries are *n (%)*

CER – cerebellum; DGM – deep grey matter; GM – grey matter; IVH – intraventricular hemorrhage; POM – pomegranate; RD – risk difference; RR – relative risk; WM – white matter

^1.^Absolute effect size calculated as the mean difference for continuous variables, and the risk difference (%) for categorical variables (Placebo = reference)

^2.^Relative effect size calculated as Cohen’s *d* for continuous variables, and the relative risk for categorical variables (Placebo = reference). For analyses with zero in one or more cells, 0.5 was added to each cell prior to calculation of the relative risk and its standard error (SE). Corresponding SE are reported.

^3.^Fisher’s exact test (2-sided) used to compare proportions by group due to cell sizes < 5.
