# Supplementary material for: Maternal pomegranate juice intake and brain structure and function in infants with intrauterine growth restriction: A randomized controlled pilot study
Source: PLoS One. 2019 Aug 21;14(8):e0219596. doi: 10.1371/journal.pone.0219596 (PMC6703683; doi:10.1371/journal.pone.0219596)
Supplement: S2 Table — (DOCX) [file pone.0219596.s006.docx]

**S2 Table.** Brain metrics by group status, adjusted for postmenstrual age at scan

|  | Group Summaries | | | | | | | | | | Group Comparisons^1^ | | | | | | | | | |
| --- | --- | --- | --- | --- | --- | --- | --- | --- | --- | --- | --- | --- | --- | --- | --- | --- | --- | --- | --- | --- |
|  | **POM**  **(n=28)** | | **POM,  Metabolite +ve**  **(n=17)** | | | **Placebo**  **(n=27)** | | **Placebo,  Metabolite -ve (n=15)** | | | **MODIFIED INTENTION-TO-TREAT** | | | | | **PER-PROTOCOL** | | | | |
|  | **Mean** | **SD** | **Mean** | **SD** | **Mean** | | **SD** | | **Mean** | **SD** | **Estimate** | **SE** | **t** | **P** | | | **Estimate** | **SE** | **t** | **P** |
| BFD | 67.46 | 3.17 | 66.88 | 3.39 | 68.26 | | 3.34 | | 67.07 | 2.91 | -0.48 | 0.79 | -0.61 | | 0.54 | | -0.05 | 1.03 | -0.04 | 0.97 |
| FH_L | 49.48 | 2.64 | 49.06 | 2.83 | 50.66 | | 2.73 | | 50.13 | 1.98 | -0.91 | 0.66 | -1.39 | | 0.17 | | -1.06 | 0.87 | -1.22 | 0.23 |
| FH_R | 49.73 | 2.70 | 49.36 | 2.86 | 51.20 | | 2.79 | | 50.67 | 2.39 | -1.23 | 0.70 | -1.77 | | 0.08 | | -1.30 | 0.96 | -1.35 | 0.19 |
| BPD | 80.82 | 4.63 | 80.88 | 5.02 | 81.26 | | 3.61 | | 80.27 | 3.41 | 0.10 | 0.89 | 0.11 | | 0.91 | | 0.92 | 1.09 | 0.84 | 0.41 |
| BBPD | 83.11 | 4.83 | 83.00 | 4.96 | 83.11 | | 3.64 | | 82.27 | 3.39 | 0.54 | 0.92 | 0.59 | | 0.56 | | 1.03 | 1.13 | 0.91 | 0.37 |
| IHD^2^ | 2.30 |  | 2.30 |  | 2.30 | |  | | 2.30 |  | -0.37 | 0.37 | -0.99 | | 0.33 | | -0.12 | 0.38 | -0.32 | 0.75 |
| CIOD_L^2^ | 1.70 |  | 2.00 |  | 2.00 | |  | | 2.00 |  | -0.30 | 0.24 | -1.27 | | 0.21 | | 0.00 | 0.35 | 0.00 | 1.00 |
| CIOD_R | 2.08 | 0.60 | 2.14 | 0.61 | 2.01 | | 0.57 | | 2.01 | 0.54 | 0.07 | 0.16 | 0.42 | | 0.68 | | 0.14 | 0.21 | 0.65 | 0.52 |
| FOHR | 0.36 | 0.02 | 0.36 | 0.02 | 0.36 | | 0.02 | | 0.37 | 0.02 | 0.00 | 0.00 | -0.49 | | 0.62 | | 0.00 | 0.01 | -0.49 | 0.63 |
| TCD | 51.23 | 3.18 | 50.91 | 3.54 | 51.04 | | 2.58 | | 51.48 | 2.06 | 0.56 | 0.62 | 0.91 | | 0.37 | | -0.36 | 0.72 | -0.49 | 0.63 |
| LVD_L | 5.98 | 1.14 | 5.89 | 1.02 | 5.88 | | 1.01 | | 5.95 | 1.13 | 0.12 | 0.29 | 0.42 | | 0.68 | | -0.06 | 0.39 | -0.16 | 0.88 |
| LVD_R^2^ | 5.60 |  | 5.70 |  | 5.50 | |  | | 5.50 |  | 0.10 | 0.41 | 0.24 | | 0.81 | | -0.56 | 0.64 | -0.87 | 0.39 |
| DNGM_L | 5.71 | 0.42 | 5.66 | 0.46 | 5.92 | | 0.29 | | 5.85 | 0.23 | -0.17 | 0.09 | -1.95 | | 0.06 | | -0.17 | 0.11 | -1.56 | 0.13 |
| DNGM_R | 5.84 | 0.40 | 5.80 | 0.44 | 5.99 | | 0.26 | | 5.93 | 0.21 | -0.12 | 0.08 | -1.46 | | 0.15 | | -0.10 | 0.11 | -0.98 | 0.33 |
| DNGM_total | 11.55 | 0.81 | 11.47 | 0.89 | 11.91 | | 0.53 | | 11.78 | 0.43 | -0.29 | 0.16 | -1.74 | | 0.09 | | -0.27 | 0.21 | -1.29 | 0.21 |

BBPD – bone biparietal diameter; BPD – biparietal diameter; BFD – bifrontal diameter; CIOD_L - left craniocaudal interopercular distance; CIOD_R - right craniocaudal interopercular distance; DNGM_L – left deep nuclear grey matter area; DNGM_R – right deep nuclear grey matter area; DNGM_total - total deep nuclear grey matter area; FH_L – left frontal height; FH_R – right frontal height; FOHR - frontal-occipital horn ratio; IHD – interhemispheric distance; LVD_L – left lateral ventricle diameter; LVD_R – right lateral ventricle diameter; POM – pomegranate; TCD – transverse cerebellar diameter

^1^Analyses run using generalized linear models (GLM) adjusted for postmenstrual age at scan

^2^ Distribution skewed (> |0.8|). Analyses run using ln transformed variables. Group summary values reflect medians, not means, of the raw distribution
