# Supplementary material for: Maternal pomegranate juice intake and brain structure and function in infants with intrauterine growth restriction: A randomized controlled pilot study
Source: PLoS One. 2019 Aug 21;14(8):e0219596. doi: 10.1371/journal.pone.0219596 (PMC6703683; doi:10.1371/journal.pone.0219596)
Supplement: S3 Table — (DOCX) [file pone.0219596.s007.docx]

**S3 Table.** Brain volumes by group status, adjusted for postmenstrual age at scan

|  | Group Summaries | | | | | | | | Group Comparisons^1^ | | | | | | | |
| --- | --- | --- | --- | --- | --- | --- | --- | --- | --- | --- | --- | --- | --- | --- | --- | --- |
|  | **POM**  **(n=28)** | | **POM,  Metabolite +ve**  **(n=17)** | | **Placebo**  **(n=27)** | | **Placebo,  Metabolite -ve (n=15)** | | **MODIFIED INTENTION-TO-TREAT** | | | | **PER-PROTOCOL** | | | |
|  | **Mean** | **SD** | **Mean** | **SD** | **Mean** | **SD** | **Mean** | **SD** | **Estimate** | **SE** | **t** | **P** | **Estimate** | **SE** | **t** | **P** |
| cGM | 127.95 | 21.94 | 127.21 | 21.11 | 135.57 | 20.37 | 128.36 | 14.93 | -4.55 | 4.09 | -1.11 | 0.27 | -0.05 | 5.31 | -0.01 | 0.99 |
| WM | 123.98 | 16.15 | 123.78 | 16.44 | 123.42 | 13.74 | 121.26 | 13.88 | 1.24 | 4.01 | 0.31 | 0.76 | 2.57 | 5.52 | 0.47 | 0.64 |
| CSF | 51.04 | 16.53 | 46.70 | 16.40 | 52.78 | 13.71 | 54.39 | 12.67 | -0.57 | 3.87 | -0.15 | 0.88 | -7.45 | 5.26 | -1.42 | 0.17 |
| DGM | 22.43 | 3.17 | 22.32 | 3.27 | 23.48 | 1.88 | 22.92 | 1.45 | -0.71 | 0.56 | -1.27 | 0.21 | -0.46 | 0.78 | -0.59 | 0.56 |
| Hippocampus | 2.65 | 0.71 | 2.74 | 0.72 | 2.72 | 0.71 | 2.76 | 0.69 | -0.10 | 0.19 | -0.52 | 0.60 | -0.05 | 0.24 | -0.21 | 0.84 |
| Amygdala^2^ | 1.02 |  | 1.01 |  | 0.99 |  | 0.98 |  | 0.04 | 0.09 | 0.47 | 0.64 | 0.04 | 0.14 | 0.32 | 0.75 |
| Cerebellum | 19.91 | 3.42 | 19.82 | 3.17 | 20.79 | 2.65 | 20.34 | 2.52 | -0.50 | 0.67 | -0.75 | 0.46 | -0.37 | 0.90 | -0.42 | 0.68 |
| Brainstem | 5.17 | 0.59 | 5.12 | 0.63 | 5.21 | 0.54 | 5.13 | 0.48 | -0.03 | 0.15 | -0.16 | 0.87 | -0.01 | 0.20 | -0.06 | 0.95 |
| Total tissue | 302.42 | 319.41 | 34.08 | 315.12 | 308.68 | 25.86 | 310.40 | 0.21 | -0.02 | -3.60 | 8.69 | 0.41 | 0.68 | 2.08 | 11.62 | 0.18 |
| ICV | 362.10 | 56.54 | 355.94 | 55.55 | 372.18 | 42.58 | 363.07 | 33.72 | -4.17 | 11.24 | -0.37 | 0.71 | -5.36 | 15.55 | -0.35 | 0.73 |

cGM – cortical grey matter; CSF – cerebrospinal fluid; DGM – deep grey matter; ICV – intracranial volume; POM – pomegranate; WM – white matter

^1^Analyses run using generalized linear models (GLM) adjusted for postmenstrual age at scan

^2^ Distribution skewed (> |0.8|). Analyses run using ln transformed variables. Group summary values reflect medians, not means, of the raw distribution
