# Supplementary material for: Maternal pomegranate juice intake and brain structure and function in infants with intrauterine growth restriction: A randomized controlled pilot study
Source: PLoS One. 2019 Aug 21;14(8):e0219596. doi: 10.1371/journal.pone.0219596 (PMC6703683; doi:10.1371/journal.pone.0219596)
Supplement: S4 Table — (DOCX) [file pone.0219596.s008.docx]

**S4 Table.** DTI measures by group status, adjusted for postmenstrual age at scan

|  | | Group Summaries | | | | | | | | | | Group Comparisons^1^ | | | | | | | | |
| --- | --- | --- | --- | --- | --- | --- | --- | --- | --- | --- | --- | --- | --- | --- | --- | --- | --- | --- | --- | --- |
|  |  | **POM**  **(n=24)** | | **POM,  Metabolite +ve**  **(n=13)** | | | **Placebo**  **(n=21)** | | | **Placebo,  Metabolite –ve**  **(n=12)** | | **MODIFIED INTENTION-TO-TREAT** | | | | **PER-PROTOCOL** | | | | |
| Tract | | **Mean** | **SD** | **Mean** | **SD** | **Mean** | | **SD** | **Mean** | | **SD** | **Estimate** | **SE** | **t** | **P^a^** | | **Estimate** | **SE** | **t** | **P** |
| *Right ALIC* FA | | 0.24 | 0.03 | 0.23 | 0.03 | 0.22 | | 0.03 | 0.22 | | 0.04 | 0.02 | 0.01 | 2.11 | 0.04 | | 0.01 | 0.01 | 0.47 | 0.64 |
| MD | | 1.23 | 0.06 | 1.22 | 0.05 | 1.26 | | 0.06 | 1.26 | | 0.06 | -0.03 | 0.01 | -2.43 | 0.02 | | -0.03 | 0.02 | -1.88 | 0.07 |
| AD | | 1.57 | 0.07 | 1.54 | 0.08 | 1.58 | | 0.07 | 1.58 | | 0.07 | -0.01 | 0.02 | -0.41 | 0.69 | | -0.03 | 0.02 | -1.10 | 0.28 |
| RD | | 1.06 | 0.06 | 1.06 | 0.05 | 1.10 | | 0.07 | 1.10 | | 0.07 | -0.04 | 0.01 | -2.78 | 0.01 | | -0.03 | 0.02 | -1.59 | 0.13 |
| *Left ALIC* FA | | 0.23 | 0.03 | 0.22 | 0.04 | 0.21 | | 0.02 | 0.20 | | 0.02 | 0.02 | 0.01 | 2.02 | 0.05 | | 0.02 | 0.01 | 1.57 | 0.13 |
| MD | | 1.23 | 0.06 | 1.22 | 0.05 | 1.27 | | 0.07 | 1.28 | | 0.06 | -0.03 | 0.01 | -2.55 | 0.01 | | -0.04 | 0.02 | -2.52 | 0.02 |
| AD | | 1.55 | 0.07 | 1.54 | 0.07 | 1.57 | | 0.08 | 1.57 | | 0.08 | -0.01 | 0.02 | -0.86 | 0.40 | | -0.02 | 0.02 | -0.95 | 0.35 |
| RD | | 1.07 | 0.07 | 1.07 | 0.06 | 1.11 | | 0.07 | 1.13 | | 0.06 | -0.04 | 0.02 | -2.76 | 0.01 | | -0.05 | 0.02 | -2.50 | 0.02 |
| *Right PLIC* FA | | 0.36 | 0.03 | 0.36 | 0.04 | 0.35 | | 0.03 | 0.34 | | 0.03 | 0.01 | 0.01 | 0.93 | 0.36 | | 0.01 | 0.01 | 1.27 | 0.22 |
| MD | | 1.09 | 0.03 | 1.08 | 0.03 | 1.11 | | 0.05 | 1.11 | | 0.03 | -0.02 | 0.01 | -2.34 | 0.02 | | -0.03 | 0.01 | -2.86 | 0.01 |
| AD | | 1.55 | 0.04 | 1.54 | 0.05 | 1.57 | | 0.04 | 1.56 | | 0.03 | -0.02 | 0.01 | -1.66 | 0.11 | | -0.02 | 0.02 | -0.87 | 0.39 |
| RD | | 0.86 | 0.05 | 0.85 | 0.04 | 0.89 | | 0.06 | 0.89 | | 0.04 | -0.03 | 0.01 | -1.99 | 0.05 | | -0.03 | 0.01 | -2.41 | 0.02 |
| *Left PLIC* FA | | 0.35 | 0.04 | 0.36 | 0.04 | 0.34 | | 0.04 | 0.34 | | 0.03 | 0.01 | 0.01 | 1.20 | 0.24 | | 0.01 | 0.01 | 1.01 | 0.32 |
| MD | | 1.09 | 0.04 | 1.09 | 0.04 | 1.11 | | 0.05 | 1.11 | | 0.04 | -0.02 | 0.01 | -2.13 | 0.04 | | -0.02 | 0.01 | -1.66 | 0.11 |
| AD | | 1.55 | 0.04 | 1.54 | 0.05 | 1.56 | | 0.03 | 1.55 | | 0.02 | -0.01 | 0.01 | -1.29 | 0.21 | | -0.01 | 0.02 | -0.45 | 0.66 |
| RD | | 0.86 | 0.05 | 0.86 | 0.05 | 0.89 | | 0.07 | 0.89 | | 0.05 | -0.03 | 0.01 | -1.88 | 0.07 | | -0.03 | 0.02 | -1.52 | 0.14 |
| *Right OR* FA | | 0.21 | 0.03 | 0.20 | 0.04 | 0.20 | | 0.02 | 0.20 | | 0.02 | 0.01 | 0.01 | 0.86 | 0.40 | | 0.00 | 0.01 | 0.07 | 0.94 |
| MD^3^ | | 1.49 |  | 1.48 |  | 1.55 | |  | 1.52 | |  | -0.04 | 0.03 | -1.39 | 0.17 | | -0.03 | 0.04 | -0.88 | 0.39 |
| AD^3^ | | 1.81 |  | 1.79 |  | 1.87 | |  | 1.82 | |  | -0.05 | 0.04 | -1.15 | 0.26 | | -0.05 | 0.04 | -1.42 | 0.17 |
| RD^2^ | | 1.34 |  | 1.32 |  | 1.39 | |  | 1.37 | |  | -0.02 | 0.02 | -1.12 | 0.27 | | -0.03 | 0.03 | -1.17 | 0.26 |
| *Left OR* FA | | 0.20 | 0.03 | 0.20 | 0.03 | 0.20 | | 0.02 | 0.20 | | 0.02 | 0.00 | 0.01 | 0.09 | 0.93 | | 0.00 | 0.01 | -0.43 | 0.67 |
| MD | | 1.51 | 0.08 | 1.49 | 0.08 | 1.53 | | 0.09 | 1.52 | | 0.10 | -0.02 | 0.02 | -0.91 | 0.37 | | -0.02 | 0.04 | -0.49 | 0.63 |
| AD | | 1.83 | 0.11 | 1.80 | 0.13 | 1.86 | | 0.10 | 1.84 | | 0.11 | -0.03 | 0.03 | -0.88 | 0.38 | | -0.03 | 0.05 | -0.62 | 0.54 |
| RD | | 1.35 | 0.08 | 1.34 | 0.07 | 1.37 | | 0.08 | 1.36 | | 0.10 | -0.02 | 0.02 | -0.82 | 0.41 | | -0.01 | 0.03 | -0.35 | 0.73 |
| *Right Frontal* FA | | 0.09 | 0.01 | 0.09 | 0.02 | 0.09 | | 0.01 | 0.09 | | 0.01 | 0.00 | 0.00 | -0.19 | 0.85 | | 0.00 | 0.01 | -0.69 | 0.49 |
| MD | | 1.47 | 0.08 | 1.45 | 0.07 | 1.48 | | 0.09 | 1.48 | | 0.09 | -0.01 | 0.02 | -0.60 | 0.55 | | -0.03 | 0.03 | -1.02 | 0.32 |
| AD | | 1.60 | 0.08 | 1.58 | 0.08 | 1.62 | | 0.11 | 1.62 | | 0.09 | -0.02 | 0.03 | -0.68 | 0.50 | | -0.04 | 0.04 | -1.14 | 0.27 |
| RD | | 1.40 | 0.08 | 1.38 | 0.07 | 1.42 | | 0.09 | 1.42 | | 0.09 | -0.01 | 0.02 | -0.54 | 0.59 | | -0.03 | 0.03 | -0.91 | 0.37 |
| *Left Frontal* FA | | 0.09 | 0.01 | 0.09 | 0.02 | 0.09 | | 0.01 | 0.09 | | 0.01 | 0.00 | 0.00 | 1.08 | 0.29 | | 0.00 | 0.01 | -0.10 | 0.93 |
| MD | | 1.44 | 0.08 | 1.43 | 0.09 | 1.46 | | 0.08 | 1.46 | | 0.08 | -0.02 | 0.02 | -0.67 | 0.50 | | -0.03 | 0.03 | -0.80 | 0.44 |
| AD | | 1.57 | 0.08 | 1.56 | 0.08 | 1.59 | | 0.09 | 1.59 | | 0.08 | -0.01 | 0.02 | -0.55 | 0.58 | | -0.03 | 0.03 | -0.89 | 0.38 |
| RD | | 1.38 | 0.08 | 1.37 | 0.09 | 1.40 | | 0.08 | 1.40 | | 0.08 | -0.02 | 0.02 | -0.72 | 0.47 | | -0.02 | 0.03 | -0.74 | 0.47 |
| *Right CNBD* FA | | 0.20 | 0.04 | 0.19 | 0.04 | 0.18 | | 0.02 | 0.18 | | 0.02 | 0.02 | 0.01 | 2.14 | 0.04 | | 0.01 | 0.01 | 0.73 | 0.47 |
| MD^2^ | | 1.31 |  | 1.30 |  | 1.34 | |  | 1.35 | |  | -0.02 | 0.01 | -1.39 | 0.17 | | -0.02 | 0.02 | -1.38 | 0.18 |
| AD^3^ | | 1.61 |  | 1.60 |  | 1.59 | |  | 1.60 | |  | 0.02 | 0.02 | 1.03 | 0.31 | | -0.01 | 0.03 | -0.25 | 0.80 |
| RD | | 1.18 | 0.06 | 1.18 | 0.07 | 1.22 | | 0.06 | 1.22 | | 0.05 | -0.04 | 0.02 | -1.86 | 0.07 | | -0.03 | 0.02 | -1.46 | 0.16 |
| *Left CNBD* FA | | 0.21 | 0.03 | 0.20 | 0.04 | 0.19 | | 0.02 | 0.19 | | 0.02 | 0.01 | 0.01 | 2.01 | 0.05 | | 0.01 | 0.01 | 0.57 | 0.57 |
| MD | | 1.31 | 0.04 | 1.32 | 0.05 | 1.35 | | 0.06 | 1.35 | | 0.05 | -0.03 | 0.02 | -1.81 | 0.08 | | -0.03 | 0.02 | -1.53 | 0.14 |
| AD | | 1.61 | 0.06 | 1.60 | 0.06 | 1.63 | | 0.08 | 1.62 | | 0.06 | -0.02 | 0.02 | -1.29 | 0.21 | | -0.03 | 0.02 | -1.15 | 0.26 |
| RD | | 1.17 | 0.06 | 1.18 | 0.06 | 1.21 | | 0.06 | 1.21 | | 0.05 | -0.04 | 0.02 | -1.92 | 0.06 | | -0.03 | 0.02 | -1.34 | 0.19 |
| *Right CSOV* FA | | 0.21 | 0.02 | 0.21 | 0.03 | 0.19 | | 0.03 | 0.19 | | 0.02 | 0.02 | 0.01 | 1.89 | 0.07 | | 0.02 | 0.01 | 1.59 | 0.13 |
| MD | | 1.35 | 0.08 | 1.33 | 0.09 | 1.38 | | 0.06 | 1.36 | | 0.04 | -0.03 | 0.02 | -1.40 | 0.17 | | -0.02 | 0.03 | -0.80 | 0.43 |
| AD | | 1.61 | 0.07 | 1.60 | 0.08 | 1.63 | | 0.05 | 1.61 | | 0.04 | 0.00 | 0.02 | 0.02 | 0.99 | | 0.00 | 0.03 | -0.14 | 0.89 |
| RD | | 1.22 | 0.09 | 1.20 | 0.10 | 1.26 | | 0.07 | 1.24 | | 0.05 | -0.04 | 0.02 | -2.06 | 0.05 | | -0.03 | 0.03 | -1.03 | 0.32 |
| *Left CSOV* FA | | 0.21 | 0.02 | 0.21 | 0.02 | 0.19 | | 0.02 | 0.19 | | 0.02 | 0.01 | 0.01 | 1.44 | 0.16 | | 0.02 | 0.01 | 1.74 | 0.10 |
| MD | | 1.34 | 0.07 | 1.32 | 0.07 | 1.37 | | 0.04 | 1.35 | | 0.04 | -0.03 | 0.02 | -2.10 | 0.04 | | -0.03 | 0.02 | -1.35 | 0.19 |
| AD | | 1.59 | 0.06 | 1.57 | 0.07 | 1.61 | | 0.05 | 1.59 | | 0.04 | -0.02 | 0.02 | -1.02 | 0.31 | | -0.02 | 0.02 | -1.09 | 0.29 |
| RD | | 1.21 | 0.08 | 1.19 | 0.08 | 1.25 | | 0.05 | 1.23 | | 0.05 | -0.04 | 0.02 | -2.22 | 0.03 | | -0.04 | 0.03 | -1.38 | 0.18 |
| *CC*  FA | | 0.28 | 0.05 | 0.28 | 0.04 | 0.31 | | 0.05 | 0.28 | | 0.04 | -0.03 | 0.02 | -1.69 | 0.10 | | 0.00 | 0.02 | -0.19 | 0.85 |
| MD^3^ | | 1.41 |  | 1.37 |  | 1.43 | |  | 1.43 | |  | -0.01 | 0.03 | -0.31 | 0.76 | | -0.03 | 0.05 | -0.49 | 0.63 |
| AD^2^ | | 1.84 |  | 1.78 |  | 1.91 | |  | 1.88 | |  | -0.03 | 0.03 | -1.20 | 0.24 | | -0.06 | 0.04 | -1.51 | 0.15 |
| RD | | 1.20 | 0.12 | 1.19 | 0.13 | 1.18 | | 0.14 | 1.25 | | 0.13 | 0.02 | 0.04 | 0.51 | 0.62 | | -0.07 | 0.05 | -1.23 | 0.23 |

AD – axial diffusivity; ALIC – anterior limb of internal capsule; CC – corpus callosum; CNBD – cingulum bundle; CSOV – centrum semiovale; FA – fractional anisotropy; Frontal – frontal lobe; l – left; OR – optic radiation; MD – mean diffusivity; PLIC – posterior limb of internal capsule; POM – pomegranate; r – right; RD – radial diffusivity

^1^Analyses run using generalized linear models (GLM) adjusted for postmenstrual age at scan

^2^ Distribution skewed (> |0.8|). Analyses run using ln transformed variables. Group summary values reflect medians, not means, of the raw distribution

^3^ Remained skewed after transformation. Raw variables modeled using quantile (median) regression (SAS PROC QUANTREG); group summary values reflect medians, not means, of the raw distribution
