# Supplementary material for: Maternal pomegranate juice intake and brain structure and function in infants with intrauterine growth restriction: A randomized controlled pilot study
Source: PLoS One. 2019 Aug 21;14(8):e0219596. doi: 10.1371/journal.pone.0219596 (PMC6703683; doi:10.1371/journal.pone.0219596)
Supplement: S1 File — (DOC) [file pone.0219596.s009.doc]

**Impact of maternal pomegranate intake on brain injury and placental pathology in infants with Intrauterine Growth Restriction (IUGR)**

**Investigators: Terrie Inder, Michael Nelson, George Macones**

**Washington University in St Louis**

**Background:**

Hypoxic-Ischemic injury in the newborn occurs at a rate of 1-3.5/1000 live births and carries with it a significant morbidity and mortality [1]. As many as 20% die in the neonatal period and 35% of survivors experience significant long term neurodevelopmental disability [2]. While advances in management have resulted in improved survival, morbidity has not significantly changed in the last several decades. One mechanism of neuronal injury in hypoxic ischemia is oxidative stress from inflammation and free radical production [3]. These oxygen free radicals overwhelm antioxidant defenses and results in widespread injury through lipid peroxidation. The brain, both in the adult and the neonate has been a focus of much interest given it inherent vulnerability to oxidative stress, limited antioxidant defenses, and high level of aerobic metabolism [4]. Often by the time an infant with hypoxic ischemic brain injury presents these inflammatory processes are underway making intervention difficult. Even mild hypothermia, the latest treatment modality, only affects the secondary phase of neuronal death when inflammatory processes are already underway. Therefore, the impact of any neuroprotective strategy that is started ex-utero is limited in its potential benefit. To curtail the inflammatory process and thus improve outcome from hypoxic ischemic injury an intervention would need to be initiated prior to the insult. This requires a shift in treatment strategy that focuses on the fetus rather than the ex-utero newborn.

***Criteria for neuroprotectant***

Predicting which fetuses carry the most significant risk of developing hypoxic ischemic injury is difficult and not possible prenatally. To intervene prior to injury a large number of pregnant women would have to be treated in order to incur benefit to only a handful of newborns. Therefore, the ideal neuroprotective candidate is one that can not only head off the mechanism of injury, but also meets criteria for widespread implementation. These criteria include safety to the women and their fetus, easily tolerability to ensure compliance, and economic feasibility.

***Polyphenols***

One potential neuroprotective candidate is the class of chemicals known as polyphenols. These natural antioxidants are found in a whole host of foods such as berries, nuts, grapes, chocolates, and teas. Defined as having at least one phenolic group per molecule, these diverse group of non-traditional nutrients have been studied and found protective in a whole host of human diseases ranging from cardiovascular disease and erectile dysfunction to neurodegenerative diseases such as Parkinson’s and Alzheimer’s [4-9]. Studies have shown that these polyphenols can cross the blood brain barrier and exert their effects directly on the brain [10, 11].

One of the highest polyphenol containing dietary supplements commercially available is pomegranate juice (PJ) which has been studied both in vitro and in vivo as a potential antioxidant without any proven side effects [6, 12, 13]. Studies in animal models of hypoxic ischemic injury have shown that maternal supplementation with polyphenols through pomegranate juice greatly improves neurologic injury [14, 15]. Loren et al. in 2005 showed that maternal intake of PJ during the last third of pregnancy in mice lead to a > 60% reduction in brain tissue loss following hypoxic-ischemic insult. This correlated with a decrease in overall caspase-3 activation (apoptosis) on pathology. Higher levels of ellagic acid, a polyphenic component, were found in the plasma of the intervention group, documenting placental transmission. This same research group in 2007 demonstrated that this neuroprotection is specific to polyphenols and is only present when given prior to the insult. Therefore, infants with hypoxic ischemic injury may indeed benefit from supplementation with systemic antioxidants such as polyphenols, but it would likely have to be administered prenatally before any insult occurred. Because predicting potentially at risk infants is not possible, no study to date has looked at maternal polyphenol administration as a potential neuroprotectant to at risk newborns.

***A potential model***

To properly test if in utero polyphenol administration can mediate antioxidant defenses and protect against hypoxic ischemia, one would need a model of brain injury that could be predicted prenatally. Intrauterine growth restriction (IUGR) is defined as fetal growth in utero that fails to meet its potential. The threshold for this inadequate growth varies depending on the source, but generally is defined as weight below the 10^th^ percentile of the general population for the specific gestational age of the fetus.

Fetuses with impaired intrauterine growth often suffer from long term placental insufficiency and subsequent chronic hypoxia not dissimilar from infants suffering from acute perinatal injury with subsequent hypoxic ischemic injury. Many IUGR infants tend to not tolerate labor secondary to their inability to withstand the transient drops in placental blood flow that accompanies normal labor. As a group these infants are twice as likely to have low apgar scores, twenty times more likely to suffer from encephalopathy, five times more likely to suffer perinatal stroke, and ten times more likely to die in the neonatal period [16-18]. Furthermore, these fetuses have a 2 to 3 fold increased risk of delivering prematurely placing them at additional risk of long term morbidity and mortality [19]. MR imaging in premature infants suffering from IUGR show decreased intracranial volume, abnormal diffusion of the posterior limb of the internal capsule, decreased cerebral gray matter, and reduced hippocampal volume that persists through term when compared to gestational aged matched controls [20, 21]. Magnetic resonance spectroscopy in these infants displays elevated lactate and creatine in the white matter, further indicating hypoxemic and nutritional injury. In long term neurodevelopmental follow up patients born with IUGR have an increased risk of developing cerebral palsy and continue to have deficits in learning, cognition, and attention up through adolescence [22-28]. These findings are more pronounced when focusing on the more severe end of the IUGR spectrum, those with evidence of abnormal umbilical flow (absent or reversed end diastolic flow) or birth weight < 3^rd^ percentile [29-31]. This central nervous system injury is likely mulifactorial, with mechanisms such as altered placental flow, repeated hypoxemia, elevated cortisol levels from alterations of the hypothalamic-pituitary-adrenal (HPA) axis regulation, nutritional deprivation, and acidosis after birth all contributing [18, 32]. Given that the majority of infants with IUGR are diagnosed prenatally by standardized ultrasound measurements (usually end of the 2^nd^ trimester) this population represents an ideal model for testing the impact of a prenatal neuroprotectant.

Preliminary studies in our laboratory have been undertaken in two phases of a previously approved protocol (IRB #201111063). The first phase was undertaken with healthy pregnancies in their last three weeks of pregnancy taking 8oz of pomegranate juice to ensure that it crossed the placenta. In this study, we confirmed placental transfer of polyphenols with HPLC detectable maternal and cord blood levels rising after two weeks on pomegranate juice. In addition, placenta from 4 women who received pomegranate juice in their last 2 weeks of pregnancy demonstrated decreased expression of heat shock proteins (HSP) in the pomegranate group as compared to controls. For phase II of this study (IRB #201111063) we randomized 41 mothers who were pregnant with infants with a diagnosis of IUGR by fetal ultrasound to receive 8oz pomegranate juice or 8oz apple juice. We were able to undertake MRI scans in 30 infants due to problems with preterm birth or movement on the MRI. On this analysis, the infants born to pregnant mothers who drink 8 oz of pomegranate juice daily in the last trimester of pregnancy demonstrated improved diffusion measures in the brain of their newborn infant representing more mature white matter microstructure and improved brain growth (see figures 1-6 below). Of importance, none of the mothers in the IUGR study had placental examination to determine the possible mechanism of benefit of pomegranate juice in pregnancies complicated by IUGR. This current study will fill this important gap in our knowledge.


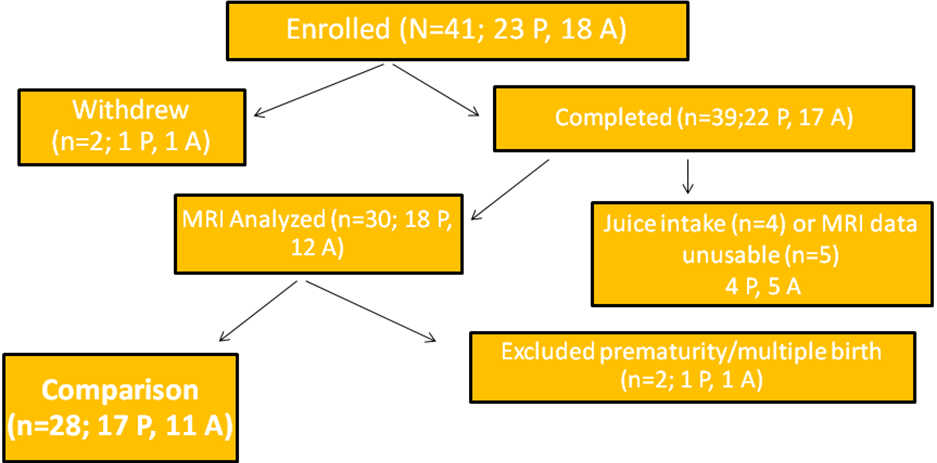
Figure 1: Pilot data in IUGR Trial in 41 mothers randomized to receive pomegranate or apple juice


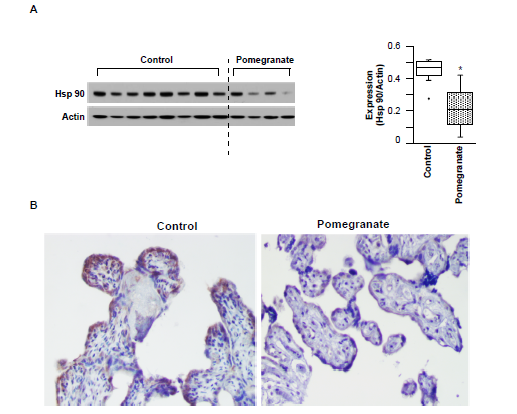

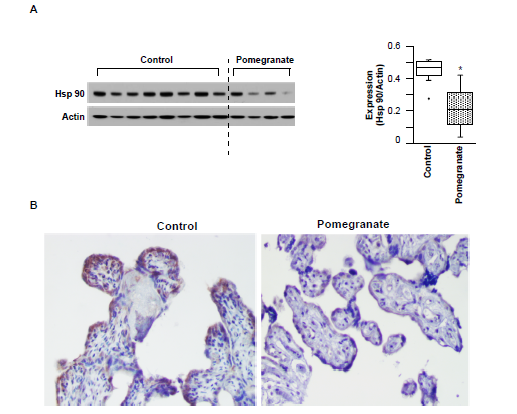
Figure 2: Placental pathology with expression for Heat Shock Protein (HSP 90) in women following labor who received 8 oz of promegranate juice compared to control in women without IUGR.

Figure 3: Cord blood levels of polyphenols at the time of birth in women in the randomized trial who received pomegranate juice (pink) or apple juice (yellow). Note the marked elevation in the levels of two polyphenols in the cord blood of infants whose mothers received pomegranate juice.


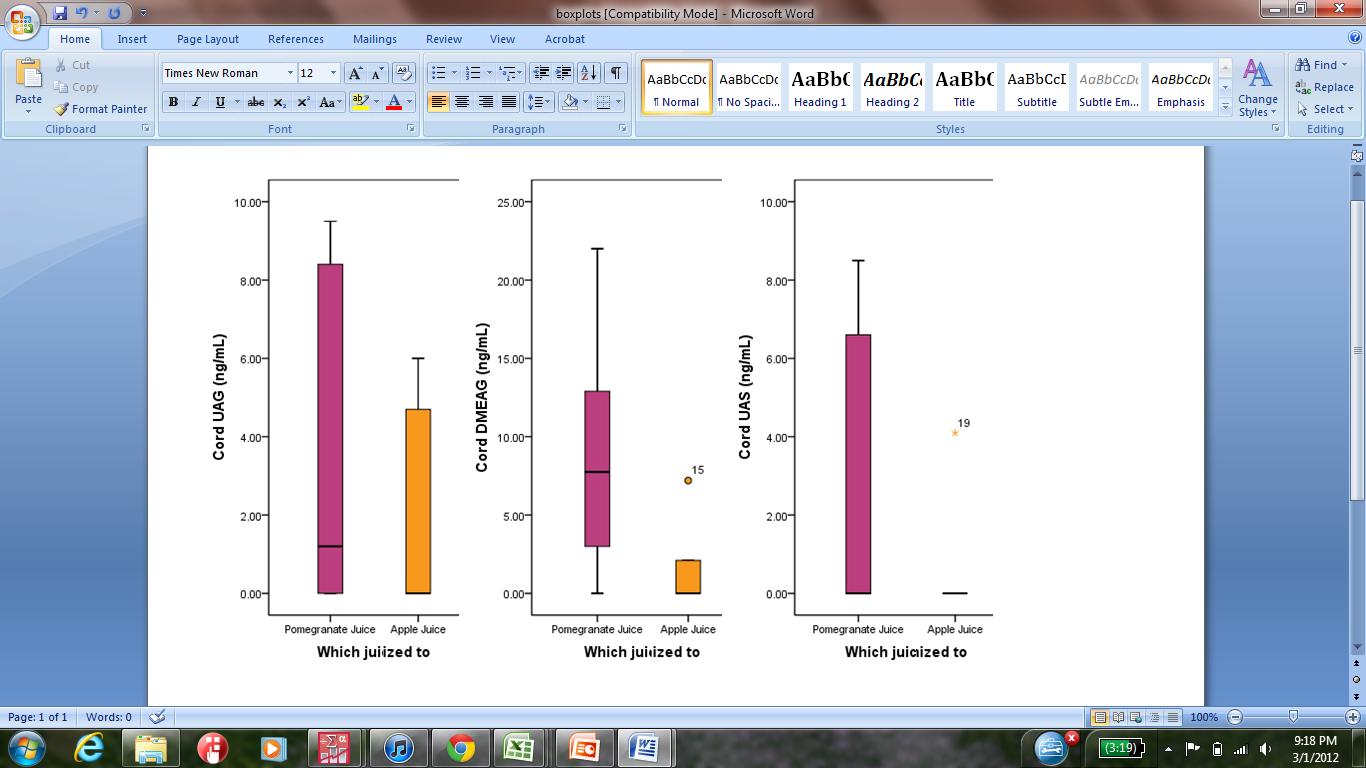

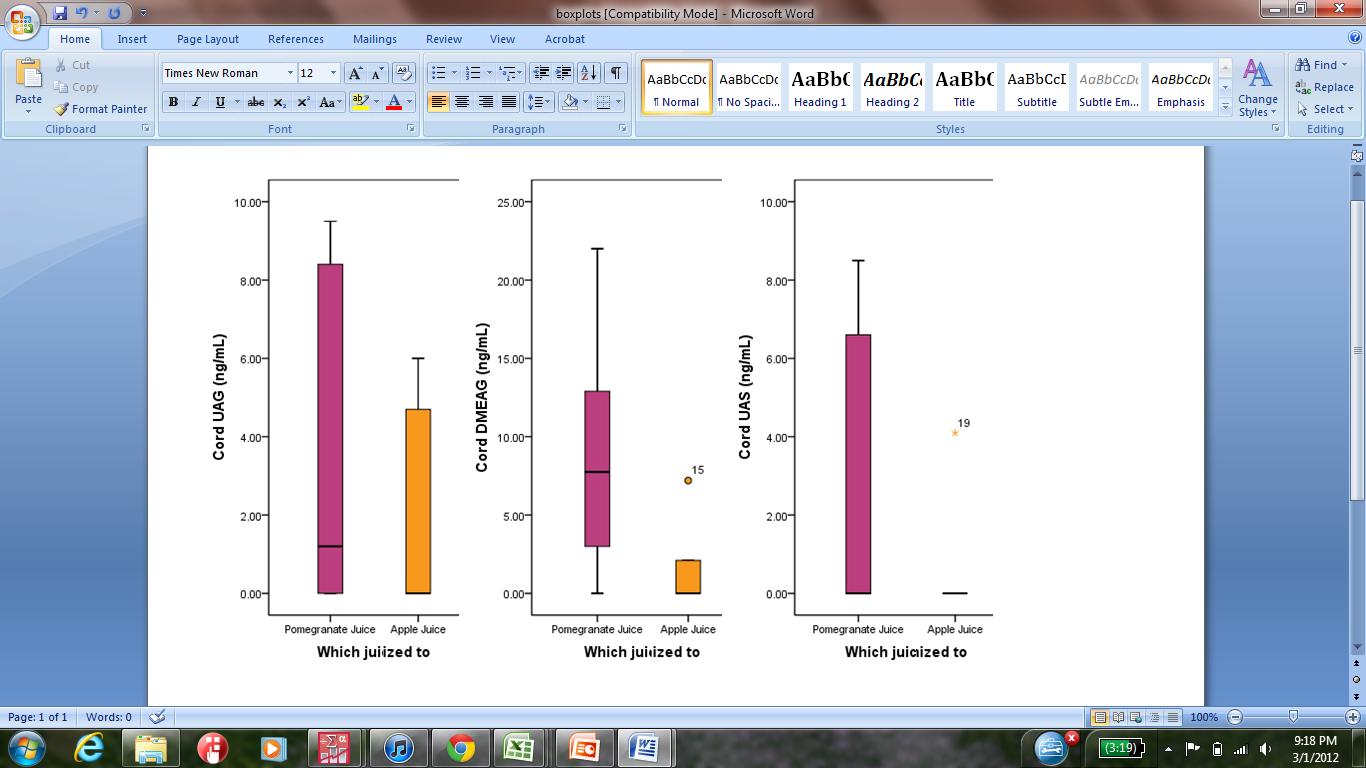


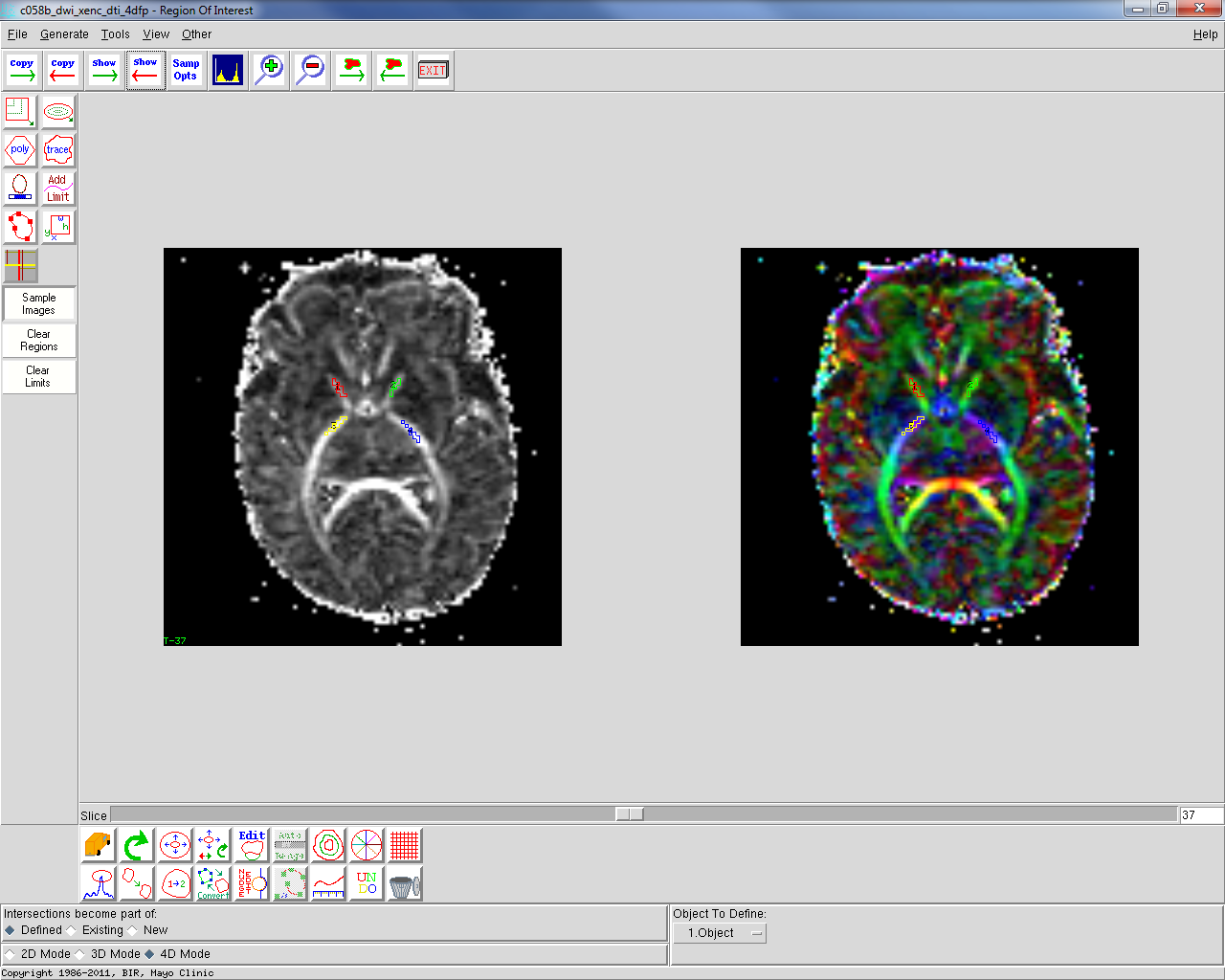

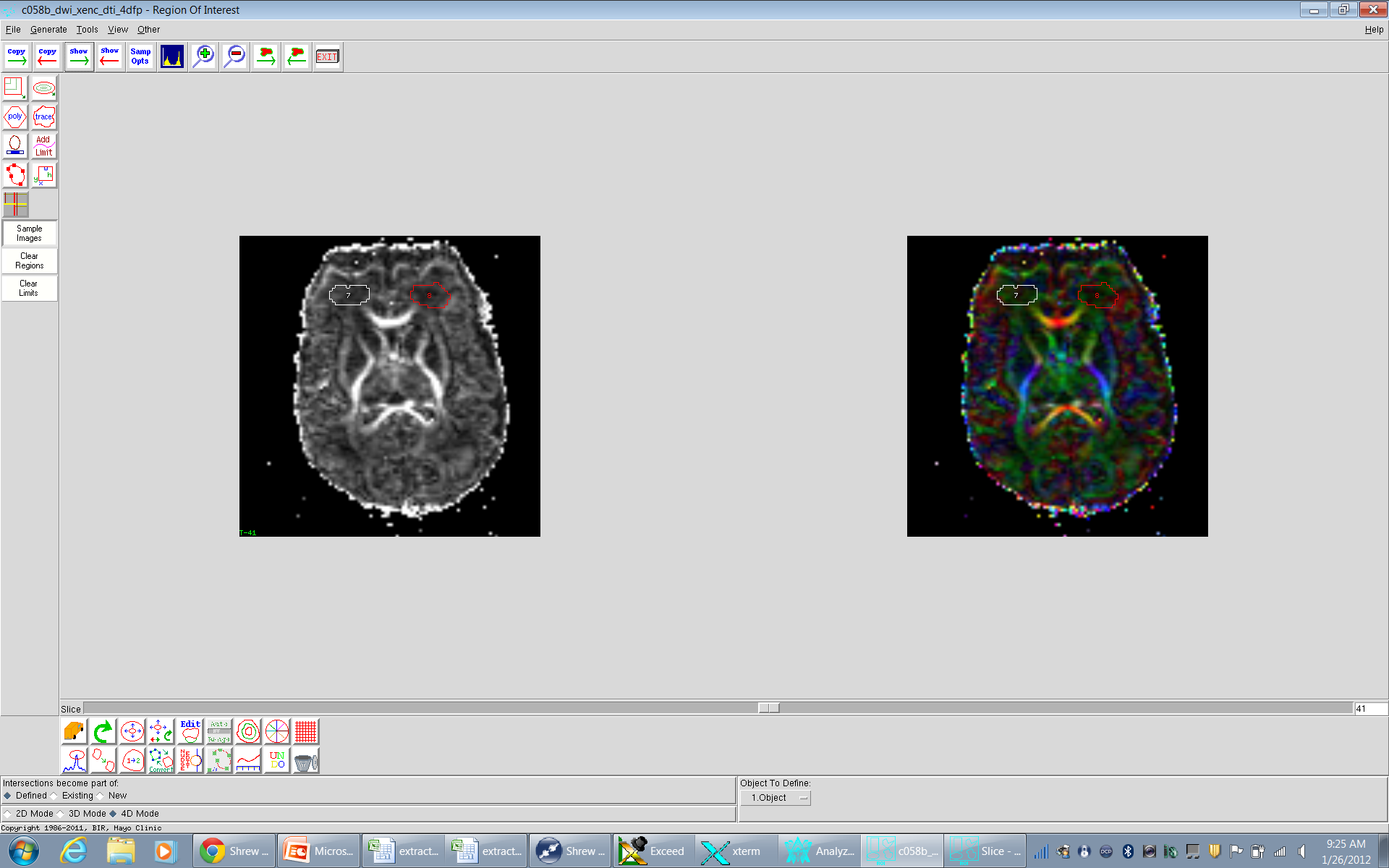
Figure 4: Brain diffusion measures were sampled in the infant brains at term equivalent. demonstrating decreased Anterior Limb of Internal Capsule diffusivity (p<0.05); increased left Frontal Lobe anisotropy (p<0.05) and trend to decreased diffusivity (p=0.07); and decreased left centrum Semiovale diffusivity (p<0.05). These changes are consistent with a more mature white matter in the infants whose mothers received pomegranate juice.

Figure 5: Brain Metrics representing brain growth demonstrated that infants who had pomegranate exposure in their mothers had larger biparietal diameters (3a) and smaller interhemispheric distances (5) and interopercular distances (4a, 4b) representing better brain growth and less extra-axial space than infants whose mothers received apple juice.


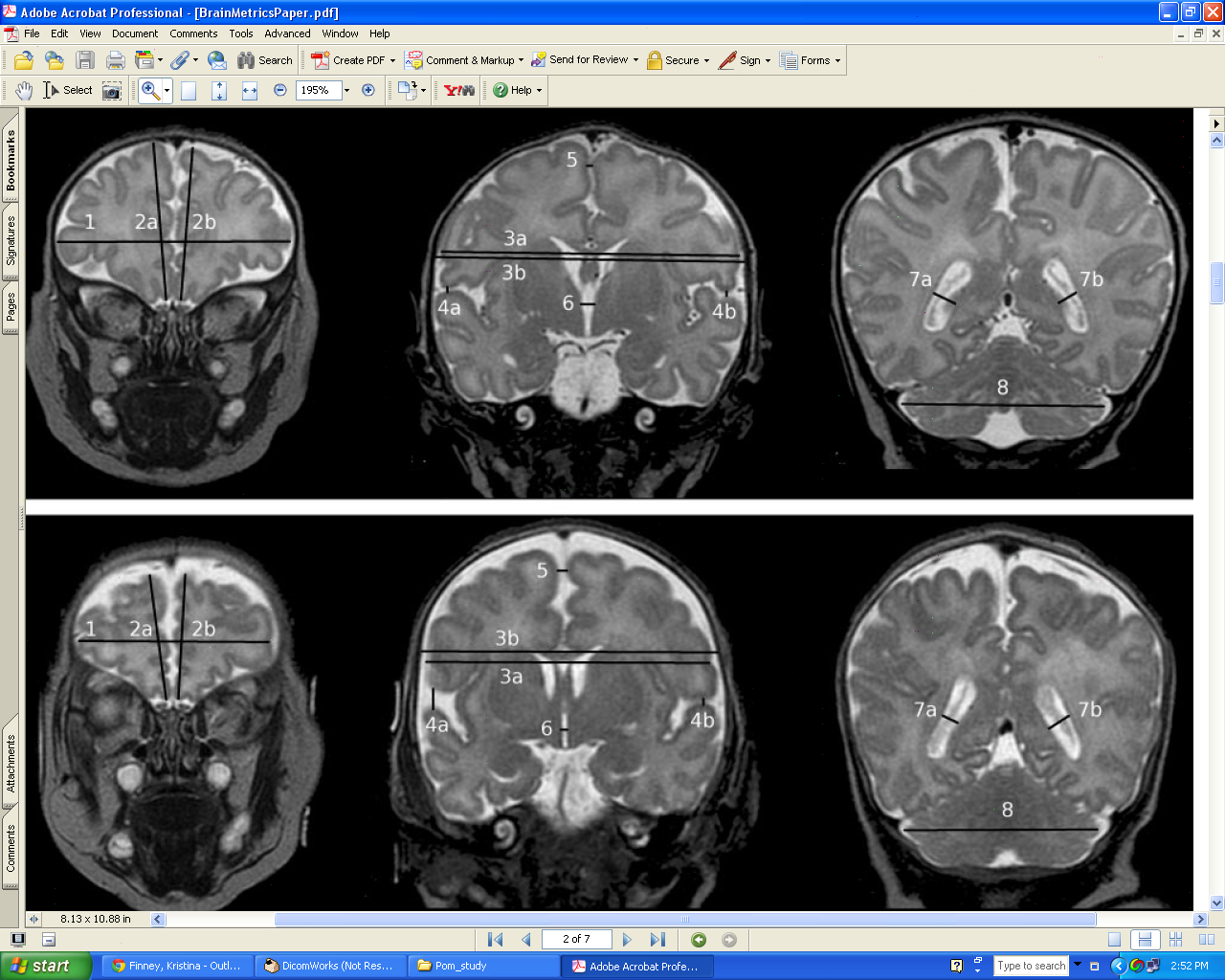


The present study seeks to investigate the impact of maternal dietary supplementation with pomegranate juice or its extract on the placenta and newborn brain development and function. This study will uniquely undertake concurrent examination of placentas and infant brain outcomes to examine the mechanism of potential benefit in IUGR.

**Hypothesis:** Maternal consumption of pomegranate juice in the last trimester of pregnancy will result in improved newborn brain development and placental function compared to infants with mothers who consume a placebo.

**Specific Aim:** To study the impact maternal polyphenol rich PJ administration has on placental function; brain injury and development (by MRI) at term and neurodevelopmental outcome at 18-24 months in infants with IUGR.

**Inclusion criteria:**

- Expecting mother with a fetal diagnosis of intrauterine growth restriction (IUGR) defined by estimated birth weight ≤ 10^th^ percentile for gestational age and gender based on the Alexander growth curves.
- ≥ 6 weeks from expected due date.

**Exclusion Criteria:**

- Multiple congenital abnormalities (other than CHD)
- Known fetal chromosomal disorder
- Maternal illicit drug or alcohol intake

**Study plan:**

Dating will be by ultrasound or reliable clinical dating by ACOG standards (33). A dedicated study coordinator will evaluate volunteers to enroll from pregnant patients at the Washington University Medical Center (WUMC) in St. Louis, MO. The Institutional Review Boards of WUMC will approve this study before initiation. A total of eighty consenting women will be randomized to one of two arms POM juice vs POM juice placebo in a 1:1 ratio. The Women’s Health Center at WUMC includes a State-approved collaborative practice of Washington University attending physicians, nurse practitioners, and residents that serve all patients regardless of payer status. The WUMC Center for Advanced Medicine offers a second site for recruitment where university affiliated private practices are located. We anticipate 1-2 patients enrolled per week.

***Treatment group****:* Expecting mothers in this group will be randomized to start a daily regimen of 8 oz glass of pomegranate juice (POM Wonderful). They will keep a daily diary documenting their compliance. They will continue this daily intake up until delivery of their infant.

***Placebo group:*** These women will start a daily regimen of an 8 oz of pomegranate free juice placebo that matches taste, calories, and appearance to regular pomegranate juice but lacks polyphenols that contains the same substances without polyphenols. They will also keep a diary of daily intake to help ensure compliance similar to the treatment group. They too will continue to take the placebos up until the time they deliver.

***Both groups:*** All women will be followed up (called) on a weekly basis to assess compliance. Upon delivery, cord blood will be collected and sent for ellagic acid, a polyphenic component. All placental material will be sent for formal pathological exam in addition to the specialized studies outlined below.

Placental Evaluation:

**Overview**: We will test the hypothesis that the active agents in pomegranate extract modulate the villous structure of the placenta, reduces the effects of exogenous stimuli, or both, in the human placenta of women diagnosed to have intrauterine growth restriction.

***Patient population:*** Placentas will be collected after delivery from all pregnancies randomized to the four study groups described above. Systematic random sampling (34, 35) of placentas midway between the chorionic and basal plate and excluding the placental edge, in area 5 B from our published work (36) will yield five specimens for formalin fixation, random orientation in paraffin blocks, and microscopic analyses below. Five separate specimens will be frozen in liquid nitrogen and prepared in Tri-Reagent for protein, RNA and DNA analysis. This number of samples controls for random variation in the placental tissue.

**Analyses to be conducted:** We will evaluate the structure, cellular composition, and expression of selected markers of oxidative stress in the placentas collected from the pregnancies of this study. W*e* have extensive experience in all aspects of the collection, morphometry, micrography, immuno- histochemistry, enzyme immunoassays, RNA and protein analyses of placental tissues.

1: Gross structure of the placenta. We will obtain the placental weight, width (short axis), breadth (long axis), thickness (average of three equidistant measurements perpendicular to the cross section of the chorionic and basal plates of the placenta), and volume of the placenta by water displacement. We also will photograph the chorionic plate to validate the cord insertion site and vascular pattern on the chorionic surface, as this is related to placental function.

2: Morphometry of villous tree: We will quantify the three types of villi (stem, intermediate, and terminal). A morphometric grid intersects tissue and cell structures to offer volume density expressed as cm^3^, while the lines that intersect tissue offer assessments of surface density, expressed as m^2^. Each placenta provides five embedded samples that are arranged in a tissue array on a single slide. Twenty digital images at 200 X final magnification are obtained from random fields and grids with a spacing of 40µm are overlain on each image for analysis. NIH Image J software (<http://rsbweb.nih.gov/ij/>) and point and line intersects mentioned above are recorded and volumes and surface areas, respectively, of placental items of interest. The morphometry analysis will quantify the relative contributions of the three villous sub-types allows us to test the hypothesis that pomegranate enhances the number of terminal villi (i.e. exchange related villi), compared to control, and that POM juice and POM extract are equal in their effects on the villous development.

3: Assessment of cellular components within villi. We will assess each villus for the relative contributions of trophoblasts by cytokeratin 7 staining, fetal blood vessels (endothelial lectin expression), connective tissue (collagen staining; macrophage immunostaining), and fibrin as a second series of evaluations to test our above hypothesis of less collagen and increased vasulature.

4: Assessment of trophoblast biology**.** *Proliferation index* in cytotrophoblasts will be evaluated by immunohistochemical staining for Ki67 antigen, which is a marker for cells that are progressing through the cell cycle. *Apoptosis* will be assessed by immunoblot analyses and immunohistochemical staining for the cl-Cyt18 and cl-Parp, as cytoplasmic and nuclear markers of apoptosis, respectively. An apoptotic index for syncytiotrophoblast and cytotrophoblasts will be calculated by dividing the fraction of positive staining cytotrophoblast by the total number of cells, or positive areas in syncytiotrophoblast normalized to surface density of this component from the morphometry evaluation. We additionally quantify the levels of cl-Cyt18 and cl-Parp by immunoblot analysis of extracts from previously frozen placental villi, with actin as a loading control. *Differentiation* will be assayed using IHC to detect hCG and hPL, both specifically produced by trophoblasts. The immunofluorescence stained expression of these hormones in syncytiotrophoblast will be quantified using confocal microscopy of tandem sections to the ones used for morphometry, as an assessment of trophoblast differentiation. Villous tissues will be evaluated by RT-PCR analysis of mRNA and western immunoblot analysis for both hCG and hPL again normalized using actin as a loading control..

5: Assessment of oxidative stress in villous tissues. We will quantify the markers of exogenous stress by immunoassay, immunohistochemistry, or western immunoblot. Each of the chosen dependent variables for this analysis assesses different components of ROS generation. *Heat shock protein 90* will be assessed by immunohistochemistry and western immunoblot analysis.

*Lipid hydroperoxide* assay will evaluate **l**ipid peroxidation in placental extracts using an assay kit (Calbiochem) with results expressed as μM/mg protein. *Nitrotyrosine assay* and immunohistochemistry for nitrotyrosine residues takes advantage of the fact that 3-nitrotyrosine, which is produced following the reaction of superoxide with nitric oxide, forms peroxynitrite which modifies tyrosine residues on proteins will be determined by commercial chemi-luminesence assay (Millipore) as μg/mg protein. *Paraoxonase 1 expression* is regulated by polyphenols in hepatocytes and this enzyme activates PPAR-γ, a key transcription factor for placental development. We will determine expression by immunoblot activity using an antibody from Sigma and the aryl esterase activity of the enzyme using phenyl acetate as substrate, reported as one unit equals 1 μmol of phenyl acetate hydrolyzed/ min. *Superoxide dismutases (SOD)* as key antioxidant enzymes will be measured by commercial assay (Caymann Chemical) that detects all three types of SOD activity (Cu-ZnSOD, MnSOD, & FeSOD). SOD activity is expressed as U/mg protein.

6: Microarray assessments of villous tissues exposed to POM juice, POM extract, or placebos. We will prepare RNA for gene microarray analyses as we have done previously (Roh et al. Placenta). We will test the hypothesis that pomegranate juice, extract or both, modulate the gene expression profile to enhance the cassette of genes involved in tissue repair, cytotrophoblast proliferation, limits expression of genes involved in apoptosis while enhancing genes involved in autophagy. This profile predicts prosurvival (e.g. increased autophagy), less cellular injury (i.e. apoptosis), and overall tissue repair (re-epithelialization of villous injury).

**Interpretation of results:** We expect pomegranate-exposed placentas to show less evidence of oxidative stress, more trophoblast mass, and less evidence of fibrin deposition microscopically and by immunoblotting. We expect less apoptosis and more evidence of trophoblast differentiation in the pomegranate group. We will consider any differences in the light of biological significance, also, as small statistically significant differences may prove relevant to the infant outcomes. Although highly unlikely based on the known effects of pomegranate, we will note any adverse effect from pomegranate extract, such as enhanced oxidative stress, increased fibrin deposition as a sign of injury, or diminished terminal villi lower than control.

Infant Neurobehavior: All infants will have a formal Dubowitz neurologic exam and an attention evaluation using the Hammersmith Neonatal Neurological Examination Optimality score at term equivalent (38-41 weeks post menstrual age). Furthermore, formal neurodevelopmental follow up will take place at 18-24 months of age.

MRI Evaluation

If clinically stable, MRI will be undertaken without sedation at term equivalent (38-41 weeks CGA). The MRI evaluation will include the following analysis:

a) Systematic qualitative scoring of the nature and extent of brain injury

b) Diffusion analysis of the cortical, white matter and posterior limb of the internal capsule to generate apparent diffusion coefficients (ADC).

c) Spectroscopy over the left basal ganglia

d) Brain metrics measuring the length of certain regions of the brain and comparing to standardized values

e) Assessment of MRI developmental indices such as volume and surface folding (SBM)

| **Measure** | **White Matter** | **Gray Matter** |
| --- | --- | --- |
| Qualitative | WM lesion score/  ventriculomegaly | Cortical and DNGM injury score |
| Metrics | Frontal lobe volume, | Intrahemispheric volume loss, ventricular size |
| Volumetry | Total myelinated and unmyelinated WM volumes | Total cortical gray matter, DNGM and CSF volumes  Hippocampal volume |
| Diffusion | Two measures (ADC, RA) each measured on 8 pairs of volume –derived lesions | Two measures (ADC, RA) on:   1. 6 pairs of surface-based regions 2. 8 pairs of volume-derived regions |
| MRS | N/A | Lactate and NAA measures over the L basal ganglia |
| SBM | NA | Four measures (cortical surface area, average sulcal depth, integrating folding index, and percentage buried cortex) |

Figure 6: Surface based renderings of prematurely born infants (five infants left side) and one term born infant (right) demonstrating marked differences in the surface brain development.


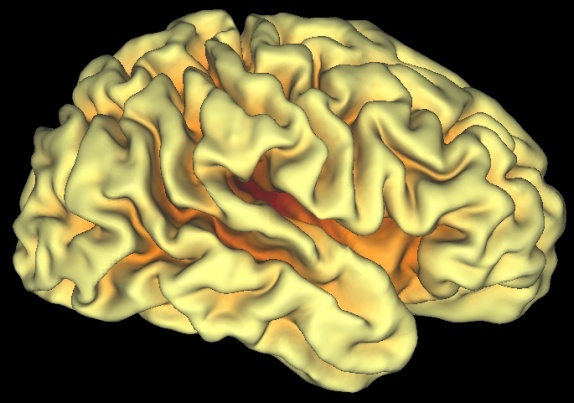

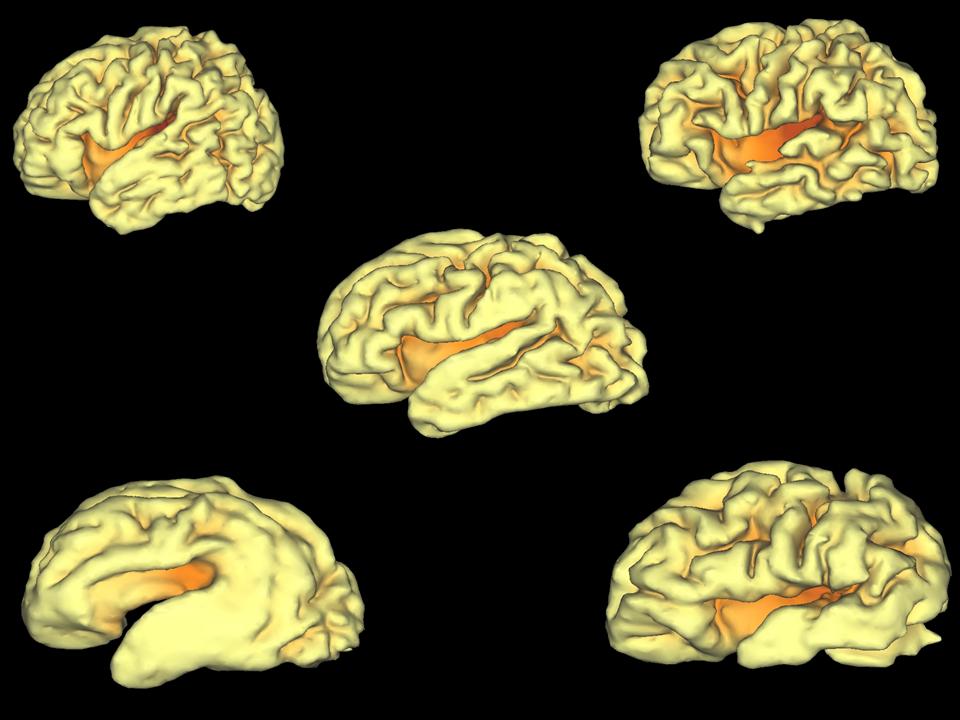


Perinatal data will also be collected. Items will include:

- Birth weight
- Gestation
- Apgars, resuscitation needed in DR and cord gas characteristics
- Any markers of neonatal illness related to admission to an intensive or special care unit such as encephalopathy, need for intravenous or parenteral nutrition or any related morbidities of preterm birth, e.g. intraventricular hemorrhage.

**Randomization:**

Mothers will be randomized at the time of enrollment by blinded envelope to either one of the treatment group or a placebo group in a 1:1 ratio.

**Outcome measures:**

1. **Placenta**

a) Morphometry – weight and size of the placenta.

b) Micrography - number of terminal villi, vasculature and collagen content.

c) Immuno- histochemistry – proliferation, apoptosis and differentiation.

d) Enzyme immunoassays - Heat shock protein 90, Lipid hydroperoxide, Nitrotyrosine assay Paraoxonase 1 expression and Superoxide dismutases

e) RNA and protein analyses of placental tissues - RNA for gene microarray analyses

1. **Pregnancy Outcomes**
   1. Complications of pregnancy – extent of pre-eclampsia, preterm labor
   2. Gestation at delivery
   3. Need for resuscitation at delivery
   4. Cord gas characteristics at delivery
2. **Brain injury and development at term by MR Imaging (see table 2) based on:**
   1. Qualitative MRI injury- white matter injury (WMI) and gray matter injury (GMI)
   2. Brain Metrics on MR imaging
   3. Diffusion- apparent diffusion coefficient (ADC) and fractional anisotropy (FA)
   4. Spectroscopy- lactate and NAA levels in the basal ganglia
   5. Advanced MRI development indices – brain volumes, surface based morphology (SBM, folding indices)
3. **Neurobehavior at term equivalent and neurodevelopmental outcome at 18-24 months** MDI and PDI by Bayley Scale of Infant Development (BSID III) (> 2SD from the norm, score ≤ 70)

***Table 1.*** Summary of the study and procedures for infants recruited into the study

| **Age** |  | **Pregnancy** | **Birth** | **38-41 weeks CGA** | **Discharge** | **18-24 months** |
| --- | --- | --- | --- | --- | --- | --- |
| Maternal data |  | X | X |  |  |  |
| Clinical data |  |  | X | X | X | X |
| Ellagic acid |  |  | X |  |  |  |
| MRI imaging |  |  |  | X |  |  |
| Neurodevelopmental  Behavioral measures | Neurologic/  Sensory |  |  | Dubowitz | Hearing |  |
|  | Attention |  |  | HNNE  attention |  |  |
|  | Global cognition |  |  |  |  | BSID-III  Cognitive |
|  | Motor |  |  |  |  | BSID-III  Motor, Neuro |
|  | Language |  |  |  |  | BSID-III  Language |

* HNNE- Hammersmith Neonatal Neurological Examination Optimality Score

* MRS- MR Spectroscopy; SBM- Surface-based morphology

**Biostatistical Analyses**

**General methods:**

The statistical methods employed in this study will be complex and will be undertaken with the assistance of Michael Wallendorf, biostatistician in the Department of Pediatrics. Clinical data such as maternal risk factors, birth weight, placental weight, gestation, need for resuscitation in the DR, etc. will be collected for both the intervention and the control group during the perinatal course.

The primary outcomes will be either a) brain injury or b) delayed development at 18-24 months. The relationship of placental function and measures to these outcomes will be explored to understand the mechanism of neuroprotection.

Brain injury will be defined as any of the following (see Table 3):

- Moderate to severe injury by qualitative scoring of WMI or GMI
- Diffusion scores (ADC, FA) > 2 SD from normal term infants
- Spectroscopy showing elevated lactate and decreased NAA (hypoxic injury) over the L basal ganglia
- Bottom 25% percentile in brain metrics, volumetric indices, or measures of surface based morphology (SBM) compared to the general healthy population.

Delayed development will be classified as:

- BSID III (MDI or PDI) > 2 SD (< 70) below the normal population

* MRS- MR Spectroscopy; SBM- Surface-based morphology

Table 2. Definition of CNS Injury by US/ MRI

| **Modality** | **Technique** | **Measures** | **Likelihood of injury** | **Definition of**  **Injury** |
| --- | --- | --- | --- | --- |
| Ultrasound |  | Grade IVH | Low | Grade 2-4 |
| MRI | Qualitative | WMI, GMI | Low | Mod-Severe injury |
|  | Diffusion/Spect | ADC, FA  Lactate, NAA | High  Low | > 2 SD above term  Lactate ↑, ↓NAA |
|  | Metrics | Intraparietal diameter, extra cranial space | High | Bottom 25% of the population |
|  | Volumetric | Total volume,  Cerebral gray matter volume, | High | Bottom 25% of the population |
|  | SBM | Cortical surface area, average sucal depth, integrated folding index | High | Bottom 25% of the population |

Continuous variables will be compared using a student t-test when they are parametric and Mann-Whitney test when they are nonparametric. Categorical variables (such as moderate or severe white matter injury) will be compared using either a chi-squared (for parametric) or a Kruskal-Wallis test for nonparametric one way analysis of variance. This is a pilot study where the results will assist in the planning of a larger randomized controlled trial.

**References:**

1. Volpe, J., ed. *Neurology of the newborn, 4th edn.* 2001, Saunders, Philadelphia.

2. Tekgul, H., et al., *The current etiologic profile and neurodevelopmental outcome of seizures in term newborn infants.* Pediatrics, 2006. **117**(4): p. 1270-80.

3. Kumar, A., et al., *Oxidative stress in perinatal asphyxia.* diatr Neurol, 2008. **38**(3): p. 181-5.

4. Aquilano, K., et al., *Role of Nitric Oxide Synthases in Parkinson's Disease: A Review on the Antioxidant and Anti-inflammatory Activity of Polyphenols.* Neurochem Res, 2008.

5. Forest, C.P., H. Padma-Nathan, and H.R. Liker, *Efficacy and safety of pomegranate juice on improvement of erectile dysfunction in male patients with mild to moderate erectile dysfunction: a randomized, placebo-controlled, double-blind, crossover study.* Int J Impot Res, 2007. **19**(6): p. 564-7.

6. Hong, M.Y., N.P. Seeram, and D. Heber, *Pomegranate polyphenols down-regulate expression of androgen-synthesizing genes in human prostate cancer cells overexpressing the androgen receptor.* J Nutr Biochem, 2008.

7. Bastianetto, S., S. Krantic, and R. Quirion, *Polyphenols as potential inhibitors of amyloid aggregation and toxicity: possible significance to Alzheimer's disease.* Mini Rev Med Chem, 2008. **8**(5): p. 429-35.

8. Mokni, M., et al., *Strong cardioprotective effect of resveratrol, a red wine polyphenol, on isolated rat hearts after ischemia/reperfusion injury.* Arch Biochem Biophys, 2007. **457**(1): p. 1-6.

9. Esmaillzadeh, A., et al., *Cholesterol-lowering effect of concentrated pomegranate juice consumption in type II diabetic patients with hyperlipidemia.* Int J Vitam Nutr Res, 2006. **76**(3): p. 147-51.

10. Mokni, M., et al., *Effect of resveratrol on antioxidant enzyme activities in the brain of healthy rat.* Neurochem Res, 2007. **32**(6): p. 981-7.

11. Youdim, K.A., B. Shukitt-Hale, and J.A. Joseph, *Flavonoids and the brain: interactions at the blood-brain barrier and their physiological effects on the central nervous system.* Free Radic Biol Med, 2004. **37**(11): p. 1683-93.

12. Seeram, N.P., et al., *Comparison of antioxidant potency of commonly consumed polyphenol-rich beverages in the United States.* J Agric Food Chem, 2008. **56**: 1415-22.

13. Heber, D., et al., *Safety and antioxidant activity of a pomegranate ellagitannin-enriched polyphenol dietary supplement in overweight individuals with increased waist size.* J Agric Food Chem, 2007. **55**(24): p. 10050-4.

14. West, T., M. Atzeva, D.M. Holtzman, *Pomegranate polyphenols and resveratrol protect the neonatal brain against hypoxic-ischemic injury.* Dev Neurosci, 2007. **29**: 363-72.

15. Loren, D.J., et al., *Maternal dietary supplementation with pomegranate juice is neuroprotective in an animal model of neonatal hypoxic-ischemic brain injury.* Pediatr Res, 2005. **57**(6): p. 858-64.

16. McIntire, D.D., et al., *Birth weight in relation to morbidity and mortality among newborn infants.* N Engl J Med, 1999. **340**(16): p. 1234-8.

17. Wu, Y.W., et al., *Perinatal stroke in children with motor impairment: a population-based study.* Pediatrics, 2004. **114**(3): p. 612-9.

18. Bukowski, R., et al., *Impairment of fetal growth potential and neonatal encephalopathy.* Am J Obstet Gynecol, 2003. **188**(4): p. 1011-5.

19. Lackman, F., et al., *The risks of spontaneous preterm delivery and perinatal mortality in relation to size at birth according to fetal versus neonatal growth standards.* Am J Obstet Gynecol, 2001. **184**(5): p. 946-53.

20. Tolsa, C.B., *Early alteration of structural and functional brain development in premature infants born with intrauterine growth restriction.* Pediatr Res, 2004. **56**: 132-8.

21. Lodygensky, G.A., et al., *Intrauterine growth restriction affects the preterm infant's hippocampus.* Pediatr Res, 2008. **63**(4): p. 438-43.

22. Leitner, Y., et al., *Neurodevelopmental outcome of children with intrauterine growth retardation: a longitudinal, 10-year prospective study.* J Child Neurol, 2007. **22**: 580-7.

23. Sung, I.K., B. Vohr, and W. Oh, *Growth and neurodevelopmental outcome of very low birth weight infants with intrauterine growth retardation: comparison with control subjects matched by birth weight and gestational age.* J Pediatr, 1993. **123**(4): p. 618-24.

24. Low, J.A., et al., *Association of intrauterine fetal growth retardation and learning deficits at age 9 to 11 years.* Am J Obstet Gynecol, 1992. **167**(6): p. 1499-505.

25. Hill, R.M., et al., *The effect of intrauterine malnutrition on the term infant. A 14-year progressive study.* Acta Paediatr Scand, 1984. **73**(4): p. 482-7.

26. Lagerstrom, M., et al., *School marks and IQ-test scores for low birth weight children at the age of 13.* Eur J Obstet Gynecol Reprod Biol, 1991. **40**(2): p. 129-36.

27. Lagerstrom, M., et al., *Behavior at 10 and 13 years of age for children with low birth weight.* Percept Mot Skills, 1990. **71**(2): p. 579-94.

28. Hagberg, G., B. Hagberg, and I. Olow, *The changing panorama of cerebral palsy in Sweden 1954-1970. III. The importance of foetal deprivation of supply.* Acta Paediatr Scand, 1976. **65**(4): p. 403-8.

29. O'Keeffe, M.J., et al., *Learning, cognitive, and attentional problems in adolescents born small for gestational age.* Pediatrics, 2003. **112**(2): p. 301-7.

30. Schreuder, A.M., et al., *Outcome at school age following antenatal detection of absent or reversed end diastolic flow velocity in the umbilical artery.* Arch Dis Child Fetal Neonatal Ed, 2002. **86**(2): p. F108-14.

31. Ley, D., et al., *Abnormal fetal aortic velocity waveform and minor neurological dysfunction at 7 years of age.* Ultrasound Obstet Gynecol, 1996. **8**(3): p. 152-9.

32. Gluckman, P.D. and J.E. Harding, *Fetal growth retardation: underlying endocrine mechanisms and postnatal consequences.* Acta Paediatr Suppl, 1997. **422**: p. 69-72.

33. ACOG practice bulletin. Diagnosis and management of preeclampsia and eclampsia. Number 33, January 2002. *Obstet Gynecol* 99: 159-167, 2002.

34. Mayhew TM. A stereological perspective on placental morphology in normal and complicated pregnancies. *J Anat* 215: 77-90, 2009.

35. Mayhew TM. Stereology and the placenta: where's the point? -- a review. *Placenta* 27 Suppl A: S17-25, 2006.

36. Wyatt SM, Kraus FT, Roh CR, Elchalal U, Nelson DM, and Sadovsky Y. The correlation between sampling site and gene expression in the term human placenta. *Placenta* 26: 372-379, 2005.
